# Healable and Reprocessable PETG-Based Dynamic Vinylogous Urethane Networks

**DOI:** 10.1021/acs.macromol.5c02738

**Published:** 2026-02-13

**Authors:** Chaninya Mak-Iad, José Augusto Berrocal, Georges J. M. Formon, Christoph Weder

**Affiliations:** † Adolphe Merkle Institute, 27211 University of Fribourg, Chemin des Verdiers 4, Fribourg 1700, Switzerland; ‡ NCCR Bio-inspired Materials, University of Fribourg, Chemin des Verdiers 4, Fribourg 1700, Switzerland; § Institute of Chemical Research of Catalonia (ICIQ), Barcelona Institute of Science and Technology (BIST), Av. Països Catalans, 16, Tarragona E-43007, Spain; ∥ ICREA, Pg. Lluís Companys 23, Barcelona 08010, Spain

## Abstract

The growing demand for sustainable materials calls for innovative strategies to extend the lifetime of polymers. In response to this challenge, we explored healable and reprocessable vinylogous urethane (VU) networks derived from commercially available glycol-modified polyethylene terephthalate (PETG). These materials were synthesized via Zn-catalyzed transesterification of PETG with ethylene glycol, end-functionalization of the resulting telechelics with bis-acetoacetate, and subsequent VU network formation using tris­(2-aminoethyl)­amine (TREN). The resulting networks combine high tensile strength (up to 48 MPa), high stiffness (0.9 GPa), and appreciable ductility (elongation at break up to 7%). An optimized network composition was reprocessed multiple times with minimal loss in performance and exhibits highly efficient healing behavior, recovering 95% of its original strength after 15 min at 180 °C. Overall, this work presents a simple and scalable route to transform a commercial high-performance polyester into a reprocessable and healable material that offers extended lifetime and improved sustainability.

## Graphical abstract

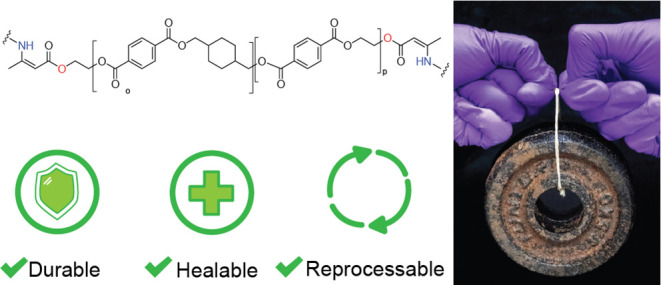

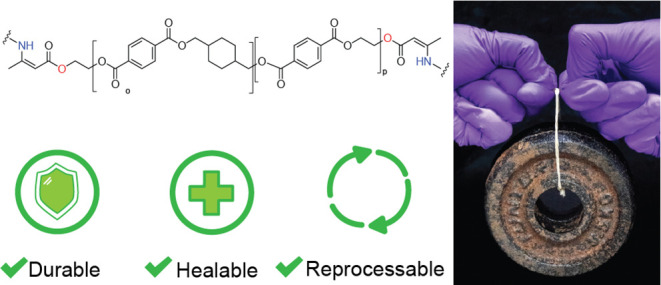

## Introduction

Self-healing polymers are an emerging class of materials capable of autonomously repairing damage or being repaired through external stimuli.^1–3^ This functionality can significantly increase the performance and extend the service life of polymers and is particularly valuable in applications where maintenance or replacement is costly or impractical, such as coatings,^4,5^ electronic skin applications,^6,7^ biomedical devices,^8,9^ and space technologies.^10,11^ Over the past two decades, several self-healing strategies have been developed,^12,13^ including intrinsically healable polymers that rely on either supramolecular interactions^14–16^ or dynamic covalent bonds.^17–19^ These reversible linkages enable polymers to reorganize at the molecular level and reform broken bonds at sites of mechanical failure, thereby restoring the original structure and function.^20–22^

Supramolecular polymers benefit from weak, reversible bonds that can dissociate under ambient conditions or exposure to specific stimuli such as heat,^23–25^ light,^26–28^ or pH changes,^29–31^ enabling rapid and efficient healing. However, these materials often suffer from limited mechanical strength or creep,^26,32,33^ unless bond dissociation is kinetically constrained (e.g., due to phase separation)^34,35^ or thermodynamically disfavored due to high association constants.^36,37^ By contrast, dynamic covalent motifs form stronger bonds that are better suited for designing robust materials with enhanced mechanical performance.^38,39^ This increased stability comes with the trade-off that healing in dynamic covalent polymer networks is generally slower than in supramolecular analogs,^40,41^ due to the higher activation barriers, as well as the reduced chain mobility that arises from the cross-linked nature of these systems.

Indeed, dynamic covalent bonds have been widely exploited to create dynamic covalent polymer networks (DCPNs), in which healing and reprocessing are possible due to structural rearrangements after bond breaking and reformation.^42–44^ In a subset of these materials, known as covalent adaptable networks (CANs),^45–47^ such rearrangements are facilitated by either dissociative or associative bond exchanges.^48–50^ In associative CANs, exemplified by vitrimers, the cross-link density remains constant, but the dynamic covalent bonds undergo degenerate exchange reactions, allowing the material to flow, be reprocessed, or heal.^51–53^ Conversely, at low temperatures, the exchange reactions are too slow and the network topology is locked in.^39,54,55^

We recently reported healable metallosupramolecular polymers (MSPs) assembled from macromonomers based on glycol-modified polyethylene terephthalate (PETG), a widely employed, amorphous polyester that offers excellent processability, high mechanical strength, and transparency,^56^ and the 2,6-bis­(′-methylbenzimidazolyl)­pyridine (Mebip) ligand.^57^ These MSPs combine high tensile strength (31 MPa) and stiffness (Young’s Modulus = 1 GPa) with excellent healability (95% strength recovery after heating 2.5 min at 160 °C), but their extensibility (3%) is limited. Motivated by the advantages of dynamic covalent strategies, we set out to explore whether incorporating reversible covalent bonds into PETG-derived networks could improve the mechanical properties vis-à-vis these MSPs, while retaining good healability and processability. We decided to explore the vinylogous urethane (VU) chemistry, pioneered by the Du Prez group, as a dynamic covalent platform for this purpose (Figure 1).^58^ VU bonds are formed through the conjugate addition of amines to acetoacetate groups with water as a byproduct, and can, in the presence of free amines, undergo exchange via transamination.^58–61^ These reactions proceed under relatively mild conditions, often without the need for external catalysts, and offer favorable kinetics for bond exchange at elevated temperatures, while the product is stable under ambient conditions. Nevertheless, it has recently been shown that CANs can be depolymerized under suitable conditions through hydrolysis based on the Le Chatelier principle.^62^ Note that the structures of the materials that we report here deviate from “typical” CANs in that they are not formed by combining low-molecular-weight monomers. Instead, one of the components is a telechelic building block with a number-average molecular weight of several kg mol^–1^ that was accessed by the controlled depolymerization of PETG.^57,63,64^ This approach not only makes the synthesis of the new materials rather straightforward, but it also leads to a structurally heterogeneous distribution of cross-links (Figure 1).

**Figure 1:**
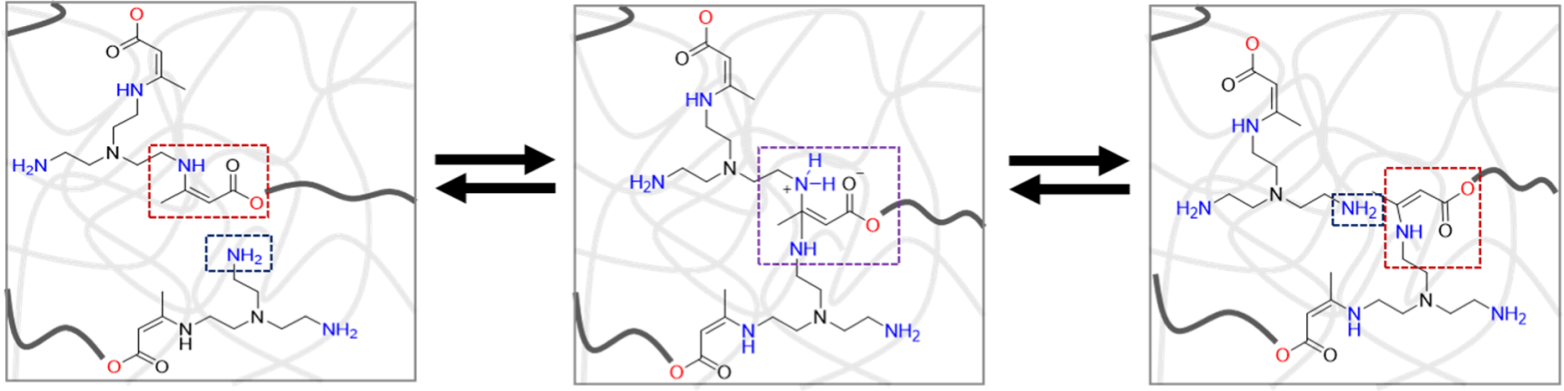
Schematic of the associative reaction mechanism in polymers featuring dynamic vinylogous urethane bonds and free amine groups.

## Experimental Section

### Materials

PETG (EASTAR 5011) was obtained from EASTMAN and dried at 80 °C in vacuo overnight before use. All other solvents and reagents were purchased from Sigma-Aldrich or Acros and were used without further purification.

### Model Reaction of Ethyl Acetoacetate with Hexylamine

In a two-necked round-bottom flask equipped with a magnetic stirrer, a septum, and a reflux condenser, hexylamine (3.84 mmol, 0.389 g, 1 equiv) and ethyl acetoacetate (3.84 mmol, 0.500 g, 1 equiv) were dissolved in CDCl_3_ (5 mL), and the reaction mixture was stirred at 60 °C under a nitrogen atmosphere. The progress of the reaction was monitored by ^1^H NMR spectroscopy. Aliquots (0.6 mL) were withdrawn after 0, 3, 6, and 12 h, transferred directly to NMR tubes, and analyzed without further purification. The conversion was determined by integrating the characteristic signals in the ^1^H NMR spectra (Figure S1).

### Model Reaction of Ethyl Benzoate with Hexylamine

In a two-necked round-bottom flask equipped with a magnetic stirrer, a septum, and a reflux condenser, hexylamine (3.84 mmol, 0.389 g, 1 equiv) and ethyl benzoate (3.84 mmol, 0.576 g, 1 equiv) were dissolved in CDCl_3_ (5 mL), and the reaction mixture was stirred at 60 °C under a nitrogen atmosphere. The progress of the reaction was monitored by ^1^H NMR spectroscopy. Aliquots (0.6 mL) were withdrawn after 0, 3, 6, and 12 h, transferred directly to NMR tubes, and analyzed without further purification. The conversion was determined by integration of characteristic signals in the ^1^H NMR spectra (Figure S2).

### Model Reaction of Ethyl Acetoacetate and Ethyl Benzoate with Hexylamine

In a two-necked round-bottom flask equipped with a magnetic stirrer, a septum, and a reflux condenser, hexylamine (4.61 mmol, 0.467 g, 1.2 equiv), ethyl benzoate (34.5 mmol, 5.19 g, 9 equiv), and ethyl acetoacetate (3.84 mmol, 0.500 g, 1 equiv) were dissolved in CDCl_3_ (5 mL), and the reaction mixture was stirred for 24 h at 60 °C under a nitrogen atmosphere. The outcome of the reaction was then probed by ^1^H NMR spectroscopy of an aliquot (0.6 mL) that was transferred to an NMR tube without purification (Figure S3).

### Synthesis of OH-Terminated Telechelic PETG (**PETG-OH**)

A bifunctional, OH-terminated **PETG** telechelic (**PETG-OH**) was prepared as reported before.^57^ SEC (THF, poly­(styrene) (PS) standard): *M*_n_ = 4221 g mol^–1^, *D̵* = 1.6; ^1^H NMR (400 MHz, CDCl_3_): *M*_n_ = 2433 g mol^–1^, δ: 8.03 (s, 22H), 4.62 (d, 13H), 4.42 (q, 2H), 4.22 (dd, 2H), 4.17–4.08 (m, 5H), 3.91 (q, 2H), 2.05–1.44 (m, 17H), 1.10 (d, 6H).

### Synthesis of PETG-Bis-Acetoacetate (**PETG-AA**)

In a 25 mL round-bottom flask equipped with a magnetic stir-bar, a septum, and a reflux condenser, the dried OH-terminated PETG telechelic (**PETG-OH**, 500 mg) was dissolved in anhydrous DMF (1 mL) under stirring at room temperature. *tert*-Butyl acetoacetate (170 mL, 5 eq. with respect to **PETG-OH**) was then added to the homogeneous solution, the resulting mixture was heated to 100 °C, and stirred at this temperature for 9 h under an inert atmosphere. After cooling to room temperature, the product was precipitated into acetone (10 mL), the precipitate was collected by filtration and washed with acetone (4 × 10 mL) and methanol (5 × 10 mL), to remove any excess *tert*-butyl acetoacetate and *tert*-butyl alcohol. The product was then dried under vacuum to yield the **PETG-AA** as a white solid (530 mg, 80%). SEC (THF, PS standard): *M*_n_ = 4704 g mol^–1^, *D̵* = 1.5; ^1^H NMR (400 MHz, CDCl_3_): *M*_n_ = 3106 g mol^–1^, δ: 8.27–8.02 (m, 30H), 4.69 (d, 18H), 4.56 (dt, 2H), 4.49 (dq, 2H), 4.29 (dd, 3H), 4.25–4.16 (m, 7H), 3.50 (d, 2H), 2.25 (d, 3H), 2.15–1.51 (m, 20H), 1.24–1.09 (m, 7H).

### Synthesis and Processing of PETG-Based Vinylogous Urethane Networks (**PETG-VU**)

**PETG-VU** networks (**PETG-VU26**, **PETG-VU37**, and **PETG-VU71**) were synthesized by reaction of **PETG-AA** with tris­(2-aminoethyl)­amine (TREN). For each material, **PETG-AA** (500 mg, 0.161 mmol, *M*_n_ = 3106 g mol^–1^, determined via ^1^H NMR spectroscopy), was charged into a 5 mL round-bottom flask equipped with a magnetic stir bar. Anhydrous dioxane (2 mL) was added, and the mixture was stirred at 80 °C until a homogeneous solution had formed. Subsequently, TREN was introduced via a micropipette; the amount was varied for each network. For **PETG-VU26**, 19.7 μL (0.135 mmol) of TREN were added, corresponding to ca. 26% excess of primary amine groups relative to the end groups in **PETG-AA** determined by ^1^H NMR spectroscopy; for **PETG-VU37**, 21.5 μL (0.147 mmol) of TREN were added, corresponding to ca. 37% excess of primary amine groups; for **PETG-VU71**, 27.6 μL (0.184 mmol) of TREN were added, corresponding to ca. 71% excess of primary amine groups. Following the TREN addition, each reaction mixture was thoroughly homogenized by stirring. The resulting solutions were cast into poly­(tetrafluoroethylene) (PTFE) Petri dishes with a diameter of 4 cm and cured and dried on a hot plate at 80 °C for 6 h under a continuous flow of nitrogen. The resulting materials were dried at 100 °C under vacuum for 12 h and subsequently hot-pressed in a Carver model 3851-0 press at 180 °C under 4 tons of pressure for 10 min between two PTFE sheets separated by 200 μm-thick spacers. After pressing, the samples were carefully removed and rapidly cooled to room temperature using cold metal plates. The resulting films had a thickness of ca. 200 μm, as measured by a micrometer, and were kept in desiccators prior to characterization. Samples made for the healing tests were prepared in the same manner, but 100 μm-thick spacers were used to prepare films with a thickness of ca. 100 μm.

### Reprocessing of PETG-Based Vinylogous Urethane Networks (**PETG-VU**)

**PETG-VU37** films were cut into small pieces, which were placed between two PTFE sheets, separated by 200 μm-thick spacers, and compression-molded in a Carver model 3851-0 press at 180 °C under 4 tons of pressure for 10 min. After pressing, the samples were carefully removed and rapidly cooled to room temperature using cold metal plates. The resulting films had a thickness of ca. 200 μm, as measured by a micrometer, and were immediately characterized after production.

### Nuclear Magnetic Resonance Spectroscopy

^1^H NMR (400 MHz) and ^13^C NMR (100 MHz) spectra were recorded on a Bruker A VIII HD spectrometer. Samples were dissolved in CDCl_3_. Spectra were processed and analyzed using MestReNova software (version 11.0). Chemical shifts (δ) are reported in parts per million (ppm) and referenced to the residual CDCl_3_ signals at δ = 7.26 ppm for ^1^H NMR and δ = 77.16 ppm for ^13^C NMR spectra. Coupling constants (*J*) are expressed in Hz. Multiplicities are denoted as follows: s = singlet, bs = broad singlet, d = doublet, dd = doublet of doublets, t = triplet, q = quartet, m = multiplet, br = broad signal. *M*_n_ values were calculated from the ratio of the integrals of the telechelics’ diagnostic backbone and end-group signals (see Figures S5 and S7).

### Size Exclusion Chromatography

SEC measurements were conducted on an Agilent Technologies 1200 series HPLC system equipped with an Agilent PLgel mixed guard column (particle size = 5 μm) and two Agilent PLgel mixed-D columns (7.5 mm ID × 300 mm L, particle size = 5 μm). THF served as the eluent at a 1.0 mL min^–1^ flow rate. Detection was achieved using a UV detector (Agilent 1200 series, λ = 346 nm) and an interferometric refractometer detector (Agilent 1260). Data acquisition and processing were carried out using Agilent ChemStation software. Number-average molecular weights (*M*_n_) and dispersity (*D̵*) values were determined relative to polystyrene (PS) standards.

### Swelling and Gel Fraction Tests

Swelling tests were performed to assess the swelling and gel fraction of the various materials by immersing samples with an initial weight (*m*_i_) of ca. 20 mg in ca. 50 mL of anhydrous THF or DMF and leaving them immersed for 24 h at room temperature. The swollen samples were weighed (*m*_s_) and then dried in a vacuum oven at 80 °C for 24 h. The dried samples were weighed (*m*_f_), and the swelling ratio and gel fraction were calculated as follows

$$Swelling\;ratio\;\left(\%\right)=\frac{m_{s}-m_{i}}{m_{i}}\times 100\%$$

$$Gel\;fraction\;\left(\%\right)=\frac{m_{f}}{m_{i}}\times 100\%$$

The values reported are averages of 3 independent measurements, and the errors quoted are standard deviations.

### Environmental Exposure at 80% Relative Humidity

Rectangular strips of **PETG-VU37** (5.35 mm wide, 0.10–0.25 mm thick) were dried in a vacuum oven at 80 °C overnight and cooled in a vacuum desiccator. The samples were then conditioned for 7 days at room temperature in an incubator at 80% relative humidity (RH), which was maintained by a saturated potassium chloride (KCl) solution in deionized water.^65^

### Thermogravimetric Analysis

TGA measurements were conducted under N_2_ atmosphere using a Mettler-Toledo TGA/DSC 1 STAR system. Samples were heated from 25 to 600 °C at a heating rate of 10 °C min^–1^.

### Differential Scanning Calorimetry

DSC analyses were carried out under a nitrogen atmosphere using Mettler-Toledo DSC 2 and DSC 5+ STAR systems. Analyses were conducted over a temperature range of −80 to 200 °C, using heating/cooling rates of 10 °C min^–1^.

### Dynamic Mechanical Analysis

DMA measurements were performed under a nitrogen atmosphere using a TA Instruments DMA Q800. Analyses were conducted over a temperature range of −80 to 200 °C, using a heating rate of 3 °C min^–1^, a frequency of 1 Hz, and an amplitude of 10 μm. Rectangular film samples (width: 5.35 mm, thickness: 0.2 mm, length: 15 mm) were used. The values reported are averages of 3–5 independent measurements, and the errors quoted are standard deviations.

### Tensile Testing

Tensile measurements were carried out at room temperature (25 °C) in accordance with ASTM D882 using a Zwick/Roell static material testing machine equipped with a 200 N Xforce HP load cell. Rectangular film samples (width: 5.35 mm, thickness: 0.2 mm, length: 15 mm) were tested at a strain rate of 150% min^–1^. The values reported are averages of 3–5 independent measurements, and the errors quoted are standard deviations.

### Rheology

Shear rheology was performed using an Anton Paar MCR 102e rheometer equipped with Peltier plates. A plate–plate geometry with 8 mm diameter and ca. 1.0−1.5 mm thick samples was used.

For **PETG-VU37**, frequency sweep experiments were conducted by first heating the sample to 180 °C, with the goal of establishing good contact with the rheometer plates, followed by rapid cooling to 100 °C. After an equilibration period of 10 min, the first measurement was carried out. Subsequent experiments at higher temperatures (120, 140, 160, and 180 °C) were carried out after heating the sample to the next higher temperature at a heating rate of 10 °C min^–1^, applying an equilibration period of 10 min, carrying out the measurement, and repeating this process. Data were collected across angular frequencies ω = 100–0.1 rad s^–1^, with a point density of five points per decade, at a constant shear strain of γ = 1%. The linear viscoelastic regime was determined using amplitude sweeps and the same temperature protocol; samples were sheared from 0.01 to 100% strain at a constant angular frequency of ω = 1 rad s^–1^.

Alternatively, for **PETG-VU37** and **PETG-VU26**, the frequency sweeps were measured by first heating the sample to 180 °C and then cooling in a stepwise manner to 160 °C, 140 °C, and 120 °C. In this case, a cooling rate of 10 °C min^–1^ was applied, but the equilibration period remained the same.

A master curve was constructed using the time–temperature superposition (TTS) principle. Frequency sweep data were shifted by manually adjusting the horizontal shift factors (*a*_T_) to superimpose the tan δ curves. The logarithms of the shift factors as a function of temperature were fitted to the Williams–Landel–Ferry (WLF) equation, with a reference temperature (*T*_R_) of 140 °C (see below), using a custom-made MATLAB code. *C*_1_ and *C*_2_ are empirical WLF constants.

$$\log a_{T}=\frac{-C_{1}\left(T-T_{R}\right)}{C_{2}+\left(T-T_{R}\right)}$$

Stress relaxation experiments followed a similar temperature protocol as the frequency sweeps. The sample was first heated to 180 °C and rapidly cooled to 120 °C to achieve good contact with the rheometer plates. After an equilibration period of 10 min, a deformation of γ = 1% was applied, and the stress was recorded over ca. 1 h. The same procedure was repeated at 130, 140, 150, 160, 170, and 180 °C. A double stretched exponential decay function (double Kohlrausch–Williams–Watts model) was fitted to the data (from 140 to 180 °C) using a custom-made MATLAB code^66,67^

$$G\left(t\right)=G_{0}^{fast}e^{{\left(-\frac{t}{\tau_{fast}}\right)}^{\beta_{fast}}}+G_{0}^{slow}e^{{\left(-\frac{t}{\tau_{slow}}\right)}^{\beta_{slow}}}$$

where *G*(*t*) is the relaxation modulus, $G_{0}^{fast}$ and $G_{0}^{slow}$ are the initial moduli, τ_fast_ and τ_slow_ are the characteristic relaxation times, and β_fast_ and β_slow_ are the stretching coefficients of the fast and slow processes, respectively.

The continuous relaxation spectra (CRS) were constructed from the data of stress relaxation experiments using the open-source Python software, pyReSpect, developed by Shanbhag.^68^ The code solves the following equation, where *H*(τ) is the continuous relaxation spectrum

$$G\left(t\right)=\int_{-\infty }^{\infty }H\left(\tau \right)e^{-\frac{t}{\tau }}d\ln\tau$$

### Scratch Healing Tests

Thin films of **PETG-VU37** (width: 5.35 mm, thickness: 0.10 mm, length: 10 mm) were used. Scratches were introduced to a depth of approximately 30% of the original sample thickness using a razor blade attached to a caliper for precise depth control. The samples were then placed in a PTFE mold placed on a heating plate heated at 180 °C for ca. 15 min, until the scratch visibly disappeared, which was confirmed by optical microscopy.

### Optical Microscopy

Images were acquired on an Olympus BX51 microscope equipped with a DP71 digital camera.

## Results and Discussions

### Model Reactions

To confirm the feasibility of selectively reacting tris­(2-aminoethyl)­amine (TREN) with the acetoacetate end groups of the building block **PETG-AA** and exclude the absence of any side reactions involving the ester backbone of the PETG telechelic, several model reactions were performed (Scheme 1). These experiments were carried out in CDCl_3_ at a reactant concentration of ca. 0.77 mol L^–1^, and the outcomes were probed by ^1^H NMR spectroscopy without any workup. In a first reaction, which was carried out under stoichiometric conditions, hexylamine was shown to readily undergo condensation with the β-keto ester ethyl acetoacetate at 60 °C under nitrogen, forming a vinylogous urethane (VU). The ^1^H NMR spectrum shows strikingly that the VU is the only product and that full conversion is reached after 6 h (Figure S1). In contrast, no conversion was observed in an attempted reaction between stoichiometric amounts of hexylamine and ethyl benzoate under identical conditions, highlighting the significantly lower electrophilicity of the ester moiety compared to the activated β-keto ester (Figure S2). This selectivity is further emphasized by a third model reaction in which hexylamine was reacted with a mixture of ethyl acetoacetate and ethyl benzoate. Even though a 9:1 ethyl benzoate to the β-keto ester ratio was used (to mimic the ratio found in the final materials, see below), the ^1^H NMR spectrum collected after 12 h shows that the only reaction product is the VU, while the slight excess of hexylamine and the ethyl benzoate remain unreacted under these conditions (Figure S3). While the direct amidation of esters such as ethyl benzoate is possible under harsher conditions,^69,70^ the model reactions reported here demonstrate clearly that they are inert under the conditions exploited here, allowing for the selective transformations of activated β-keto esters in the presence of less reactive ester functionalities.^71–73^

**Scheme 1:**
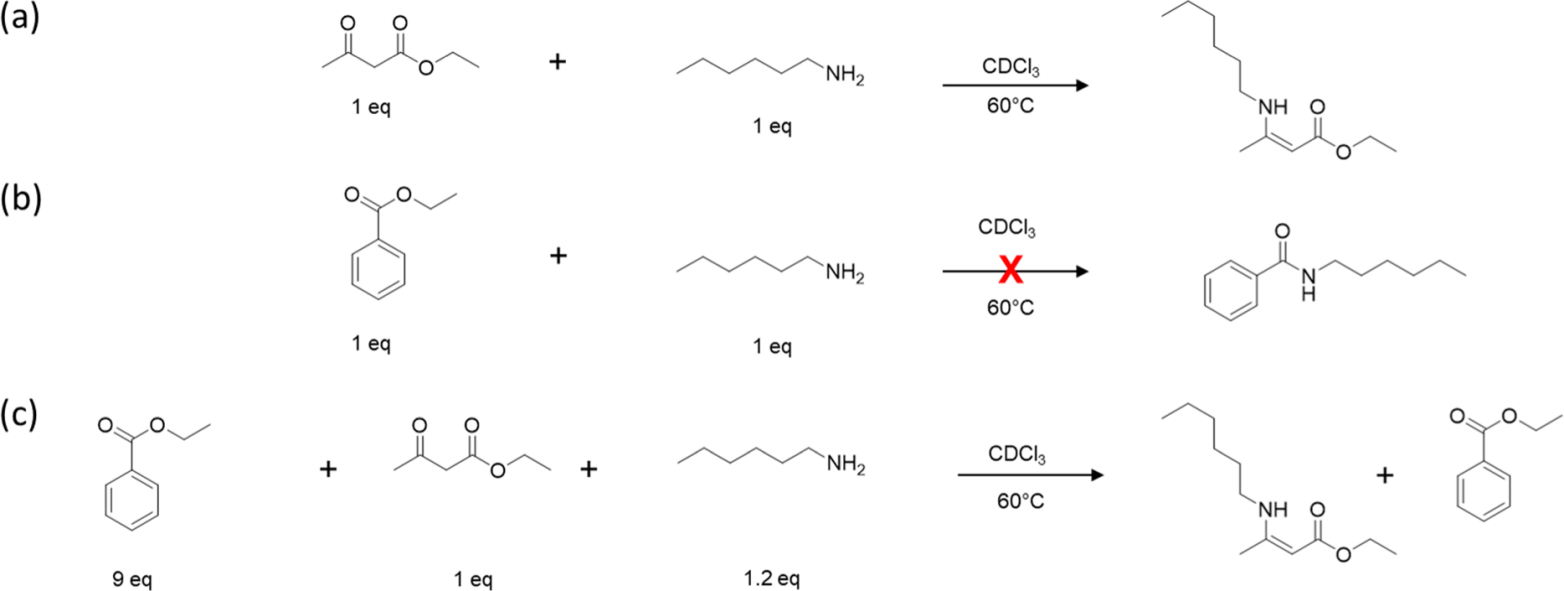
Model Reactions Were Carried out to Probe the Reactivity of Hexylamine With Ethyl Acetoacetate and Ethyl Benzoate; These Include: (a) The Reaction of Hexylamine With Ethyl Acetoacetate, (b) The Reaction of Hexylamine With Ethyl Benzoate, and (c) The Reaction of Hexylamine With a 9:1 Mixture of Ethyl Benzoate and Ethyl Acetoacetate

### Synthesis and Characterization of PETG-Based Vinylogous Urethane Networks

The initial step of the synthesis (Scheme 2) of the **PETG-VU** networks involves the controlled depolymerization of commercial PETG via glycolysis with 17.5 mol % ethylene glycol in *N*,*N*-dimethylformamide (DMF) at 100 °C, catalyzed by zinc acetate, as previously reported (Scheme S1).^57^ This transesterification reaction yields a hydroxyl-terminated PETG telechelic (**PETG-OH**), whose molecular weight is controlled by the reaction time. Size-exclusion chromatography (SEC) analysis reveals that the depolymerization conditions used here decrease the number-average molecular weight (*M*_n_) from 30 kg mol^–1^ to 4 kg mol^–1^ (SEC), while the dispersity (*D̵* = 1.6) remains unchanged (Figure S4 and Table S1). End-group analysis by ^1^H and ^13^C NMR spectroscopy (Figures S5 and S6) provides a slightly lower *M*_n_ (2.4 kg mol^–1^). This discrepancy likely arises from the inaccuracy of the SEC at low molecular weights and the use of a PS standard (Table S1). **PETG-OH** was end-functionalized through acetoacetylation with an excess of *tert*-butyl acetoacetate and thus converted into PETG-bis-acetoacetate (**PETG-AA**) (Figure S4 and Table S1).^74,75^ Excess reagents were removed by precipitation in acetone and further washing of the product with acetone and methanol. The successful incorporation of the acetoacetate groups was confirmed by ^1^H and ^13^C NMR spectroscopy (Figures S7–S9), and the *M*_n_ (3.1 and 4.7 kg mol^–1^ by ^1^H NMR and SEC, respectively) increased slightly vis-à-vis **PETG-OH**, likely due to the loss of low-molecular-weight chains during workup, which also explains the slight decrease in *D̵* measured through SEC, from 1.6 (**PETG-OH**) to 1.5 (**PETG-AA**, see Figure S4).

**Scheme 2:**
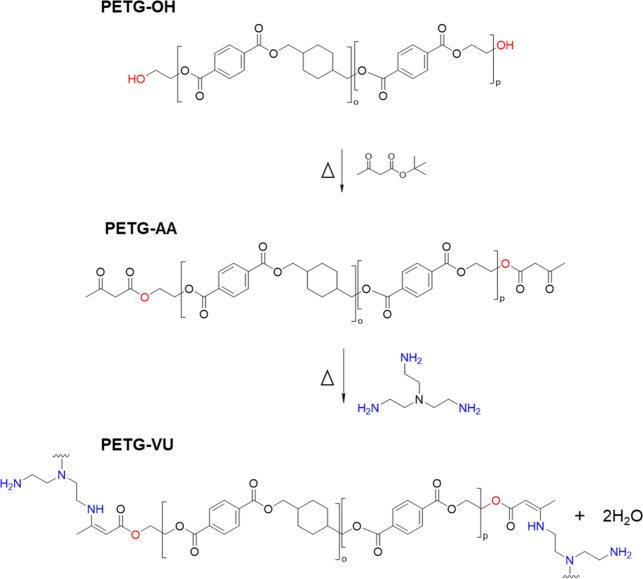
Synthesis of the Telechelic Macromonomer PETG-Bis-Acetoacetate (**PETG-AA**) and of the PETG-Based Vinylogous Urethane Dynamic Networks (**PETG-VU**)

With **PETG-AA** in hand, PETG-based vinylogous urethane networks were synthesized by reaction with TREN. **PETG-AA** was first dissolved in dioxane at 80 °C, followed by the addition of a predetermined amount of TREN; the NMR *M*_n_ of **PETG-AA** was used to establish the stoichiometry. Since the vinylogous urethane linkage is an associative dynamic covalent bond, an excess of amines is required to promote bond exchange. Accordingly, TREN was added in quantities that provide a 26% (**PETG-VU26**), 37% (**PETG-VU37**), or 71% (**PETG-VU71**) molar excess of primary amine groups relative to the acetoacetate groups in **PETG-AA**. Note, however, that the difference in weight fraction of TREN between **PETG-VU26** and **PETG-VU71** is minute (ca. 1% of the total mass). The resulting mixtures were cast into poly­(tetrafluoroethylene) (PTFE) molds, and the solvent was evaporated at 80 °C (this temperature was selected based on screening experiments in which the drying temperature was varied, see Figure S10) under a continuous flow of nitrogen. After drying, the networks were cured at 100 °C under vacuum for 12 h and subsequently compression-molded at 180 °C for 10 min. This final processing step not only improves the quality of films but also erases any crystalline domains that may have formed during preparation.^57^

Evidence for the successful condensation is provided by FT-IR spectroscopy, which reveals, in addition to the characteristic C=O stretching vibrations at 1714 cm^–1^ associated with ester groups in the backbone of the telechelic, weak but distinct absorptions at 1649 cm^–1^ and 1605 cm^–1^, corresponding to the carbonyl and C=C stretching modes of the vinylogous urethane linkages (Figure S11).^74–76^ The FT-IR spectra of all **PETG-VUs** are identical, reflecting that their compositional differences cannot be identified with this technique. To evaluate the extent of network formation, the gel fraction and swelling ratio of cured **PETG-VU** samples were measured after immersion in anhydrous THF and DMF, two solvents in which the macromonomers are fully soluble. Interestingly, **PETG-VU37** showed the highest gel fractions (63% in THF and 81% in DMF) and lowest swelling ratios (276% in THF and 94% in DMF), reflecting the highest cross-link density (Table S2). **PETG-VU26** and **PETG-VU71** both exhibited lower gel fractions (51% in THF and 74% in DMF for **PETG-VU26**; 30% in THF and 56% in DMF for **PETG-VU71**), pointing to lower cross-link densities, especially in the case of **PETG-VU71**. This is further supported by their higher swelling ratios (327% in THF and 132% in DMF for **PETG-VU26**; 648% in THF and 338% in DMF for **PETG-VU71**) (Table S2). While the low cross-link density of **PETG-VU71** is clearly driven by the large excess of amine groups, it is not immediately clear why the apparent cross-link density of **PETG-VU26** is lower than that of **PETG-VU37**.

### Thermal Properties of PETG-Based Vinylogous Urethane Networks

The thermal stability and thermal transitions of the various PETG-based materials were evaluated by thermogravimetric analysis (TGA) and differential scanning calorimetry (DSC). The original PETG and the depolymerized telechelic **PETG-OH** exhibit high thermal stability, with 5% weight-loss temperatures (*T*_d5%_) in the range of 380–390 °C (Figure S12 and Table 1). The acetoacetylated **PETG-AA** exhibits a reduced thermal stability, with a *T*_d5%_ of ca. 330 °C. The **PETG-VU** networks (**PETG-VU26**, **PETG-VU37**, **PETG-VU71**) display similar degradation temperatures (*T*_d5%_ ≈ 320 °C). Thus, all materials are stable at the temperatures utilized here for their synthesis or processing. The DSC data shows notable differences in thermal transitions across the different materials (Figure S13 and Table 1). The first and second DSC heating traces of the parent PETG show only a glass transition at a temperature (*T*_g_) of ∼84 °C, while the first heating trace of **PETG-OH** additionally reveals endothermic peaks around 108 and 165 °C, which we relate to the melting of crystalline domains^57,77,78^ whose formation is promoted by the reduced molecular weight. The second heating trace shows a significant reduction in *T*_g_ (∼66 °C) and only a weak melting peak around 180 °C, indicating that the morphology and thermal properties depend on the processing history. The DSC traces of **PETG-AA** reflect a similar behavior, although both *T*_g_ (54 °C) and melting temperature (169 °C) are slightly reduced. The DSC traces of the **PETG-VU** networks, which, in contrast to the linear PETG precursors, had been compression-molded at 180 °C, show exclusively glass transitions in the range of ∼74–82 °C with no detectable melting transitions, indicating that these materials appear to be fully amorphous.

**Table 1:** Thermal Properties of the **PETG-VU** Networks and their Precursors

sample	*T*_g,first_ ^a^ (°C)	*T*_m,first_ ^a^ ^,^ ^b^ (°C)	*T*_g,second_ ^a^ (°C)	*T*_m,second_ ^a^ ^,^ ^b^ (°C)	*T*_d5%_ ^c^ (°C)
**PETG**	80	n.a.	84	n.a.	390
**PETG-OH**	80	108, 165	66	180	380
**PETG-AA**	58	102, 141, 176	54	169	330
**PETG-VU26**	71	n.a.	82	n.a.	318
**PETG-VU37**	75	n.a.	80	n.a.	321
**PETG-VU71**	74	n.a.	74	n.a.	322

a Glass transition (*T*_g_) and melting (*T*_m_) temperatures were determined by DSC at a heating rate of 10 °C min^–1^. The data shown were extracted from the 1st and 2nd heating traces as indicated.

b Temperatures quoted correspond to the maxima of the transition; n.a. = not applicable.

c 5% weight-loss temperature determined by TGA at a heating rate of 10 °C min^–1^.

### Mechanical Properties of Vinylogous Urethane Networks

The mechanical properties of the **PETG-VU** networks were evaluated using dynamic mechanical analysis (DMA) and uniaxial tensile tests (Figure 2 and Table 2). The DMA traces reveal that all networks exhibit a high storage modulus (*E*′) of ca. 1.0 GPa at 25 °C, consistent with their glassy nature at this temperature (Figure 2a). The glass transition temperatures, determined from the peaks of the tan δ curves, are around 80 °C for all networks and closely match the *T*_g_ values determined by DSC. Above the *T*_g_, the materials display divergent thermomechanical behavior. The DMA traces of **PETG-VU26** and **PETG-VU37** feature well-defined, although slightly sloped, rubbery plateaus extending to 188 and 207 °C, respectively, indicative of load-bearing networks not far from the gel point. At *T*_g_ +40 °C, 123 and 120 °C for **PETG-VU26** and **PETG-VU37**, respectively, the cross-link density of the networks was calculated to be 74 and 100 mol m^–3^ (using the equation *d* = *E*′/3*RT*, considering an ideal Poisson ratio of 0.5), further highlighting the low degree of cross-linking.^67^ In contrast, the DMA trace of **PETG-VU71** shows a sharp drop in modulus post-*T*_g_ and a markedly lower failure temperature of 137 °C. The absence of a rubbery plateau in **PETG-VU71** aligns well with the large excess of amine groups, which limits the cross-link density, as also reflected by the low gel fraction (30% in THF and 56% in DMF).

**Figure 2:**
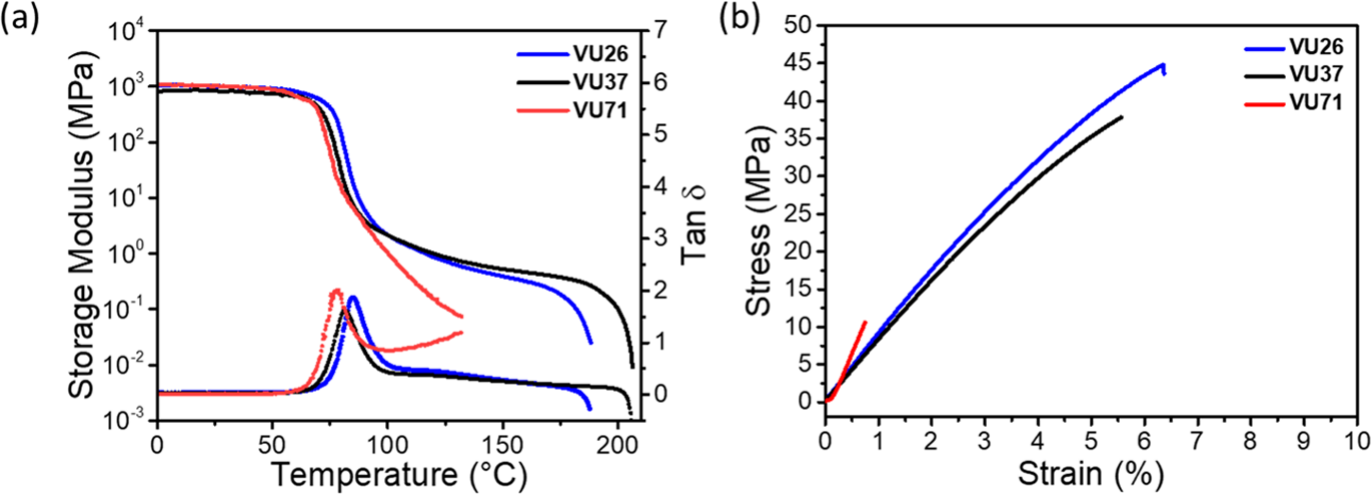
Mechanical properties of the **PETG-VU**s. (a) Dynamic mechanical analysis (DMA) traces showing the storage modulus *E*′ and loss factor tan δ. (b) Representative stress–strain curves. DMA experiments were carried out at a heating rate of 3 °C min^–1^, and tensile tests were conducted at 25 °C and a strain rate of 150% min^–1^.

**Table 2:** Thermal and Mechanical Properties of the **PETG-VU**s^a^

sample	*T*_g_ (°C)^b^	*E*′ at 25 °C (MPa)^b^	*E*′ at 150 °C (MPa)^b^	failure temp. (°C)^b^	Young’s modulus (MPa)^c^	tensile strength (MPa)^c^	strain at break (%)^c^	toughness (kJ m^–3^)^c^
**PETG-VU26**	83 ± 2	998 ± 94	0.46 ± 0.07	188 ± 2	889 ± 45	48 ± 4	7 ± 2	213 ± 53
**PETG-VU37**	80 ± 2	1069 ± 203	0.60 ± 0.15	207 ± 8	838 ± 19	40 ± 2	6 ± 1	148 ± 28
**PETG-VU71**	81 ± 6	1092 ± 106	-	137 ± 9	962 ± 47	13 ± 2	1 ± 0.2	6 ± 2

a All data represent averages of *n* = 3 individual measurements ±standard deviation.

b Glass transition temperatures (*T*_g_), storage moduli (*E*′), and failure temperatures were determined by DMA at a heating rate of 3 °C min^–1^.

c Measured by tensile tests at 25 °C and a strain rate of 150% min^–1^.

Tensile tests complete the mechanical profiles of the VU networks (Figure 2b). All three networks exhibit a high Young’s modulus (0.8–1 GPa) that confirms the high stiffness reflected by DMA. The fact that at 25 °C the three materials exhibit *E*′ values and Young’s moduli that are statistically indifferent is consistent with the fact that in glassy polymers, the modulus is primarily determined by segmental stiffness and packing, rather than network elasticity. **PETG-VU71** is rather brittle and displays by far the lowest tensile strength (13 MPa) and strain at break (1%) of the materials investigated. **PETG-VU26** and **PETG-VU37** exhibit superior mechanical performance, with tensile strengths of 48 and 40 MPa and elongations at break of 7% and 6%, respectively. These properties translated to toughness values of 213 and 148 kJ m^–3^ for **PETG-VU26** and **PETG-VU37**, respectively. Intriguingly, both **PETG-VU26** and **PETG-VU37** exhibit a higher tensile strength than the parent PETG (ca. 33 MPa, Figure S14, and Table S4), which yields at a strain of ca. 4%, and fails at a strain of ca. 242%.

### Rheological Properties of **PETG-VU**

Due to the poor thermomechanical properties and brittleness of **PETG-VU71**, rheological investigations were limited to the more robust **PETG-VU26** and **PETG-VU37** networks. These samples were subjected to oscillatory shear rheology to assess their viscoelastic behavior and dynamic bond exchange at elevated temperatures. For **PETG-VU26**, multitemperature frequency sweeps were performed by cooling the sample from 180 °C (Figure S15). At this temperature, the material exhibits viscous behavior, with the loss modulus (*G*″) exceeding the storage modulus (*G*′) across most of the accessible frequency range. Upon cooling to 160 °C, the material stiffens as expected; at lower angular frequencies (after ca. 3.5 min of experiment time), both *G*′ and *G*″ increase sharply, indicating an apparent structural transformation. Continued cooling further accentuates this stiffening, with a decrease in the loss factor (tan δ) to below 0.3, which is indicative of the sample solidifying. This is likely due to the onset of previously reported annealing-induced crystallization of the PETG segments,^57,77,78^ which can be facilitated by dynamic exchange of cross-links,^79–81^ as supported by the appearance of a distinct melting peak in the DSC trace of a **PETG-VU26** sample after rheological testing (Figure S16). This temperature-driven crystallization complicates the rheological characterization of **PETG-VU26** and must also be considered when evaluating thermal (re)­processing and healing. In this context, we note that crystallization is slow, and all solid **PETG-VU26** samples investigated here were fully amorphous, as confirmed by DSC (Figure S13 and Table 1).

In the case of **PETG-VU37**, frequency sweep experiments were also conducted by cooling the sample from 180 to 100 °C (Figure S17). *G*′ remains higher than *G*″ throughout the temperature range, and both gradually increase with decreasing temperature. The tan δ values stay between 0.3 and 0.5, except in the high frequency region at 100 °C, where they rose to 0.74 at an angular frequency (ω) of 100 rad s^–1^, typical of materials nearing the *T*_g_. However, time–temperature superposition (TTS) failed, possibly due to side reactions that can occur at high temperatures. This prompted a change in protocol: the sample was first heated to 180 °C to ensure adhesion to the rheometer plates, followed by rapid cooling to 100 °C. Amplitude sweeps were performed by heating the sample gradually from 100 to 180 °C. They reveal a linear viscoelastic regime that remains stable up to 10% strain across this temperature range (Figure S18). Frequency sweeps were then performed following the same temperature protocol at a fixed strain of γ = 1%, well within the linear regime (Figures 3a and S19a,b). Also with this protocol, *G*′ exceeds *G*″ across the entire temperature range, with gradual softening, marked by the decrease of the moduli, with increasing temperature. As also observed in the DMA trace (Figure 2a), the value of *G*′ in the rubbery regime is not constant, as would be expected for a permanent network, but instead slightly decreases with decreasing frequencies and increasing temperature, indicating increasingly dynamic behavior and an incomplete formation of the network. The tan δ values remain low (0.16–0.66) throughout the experiment (Figure S19b), indicating a rubber-like response across the tested temperature range, which is consistent with the DMA data. Application of the time–temperature superposition (TTS) principle enabled the construction of master curves by manually shifting the tan δ traces to obtain horizontal shift factors (*a*_T_) (Figures S19c,d). The drop in moduli observed at 180 °C could be indicative of the emergence of side reactions. Notably, *a*_T_ decays rapidly with increasing temperature, in a manner that follows the Williams–Landel–Ferry (WLF) equation (Figure S19e).^82^ This behavior indicates that the frequency sweep experiments capture the segmental dynamics of the network and that the *T*_g_ is superior to the topology freezing temperature (*T*_v_).^67,83^

**Figure 3:**
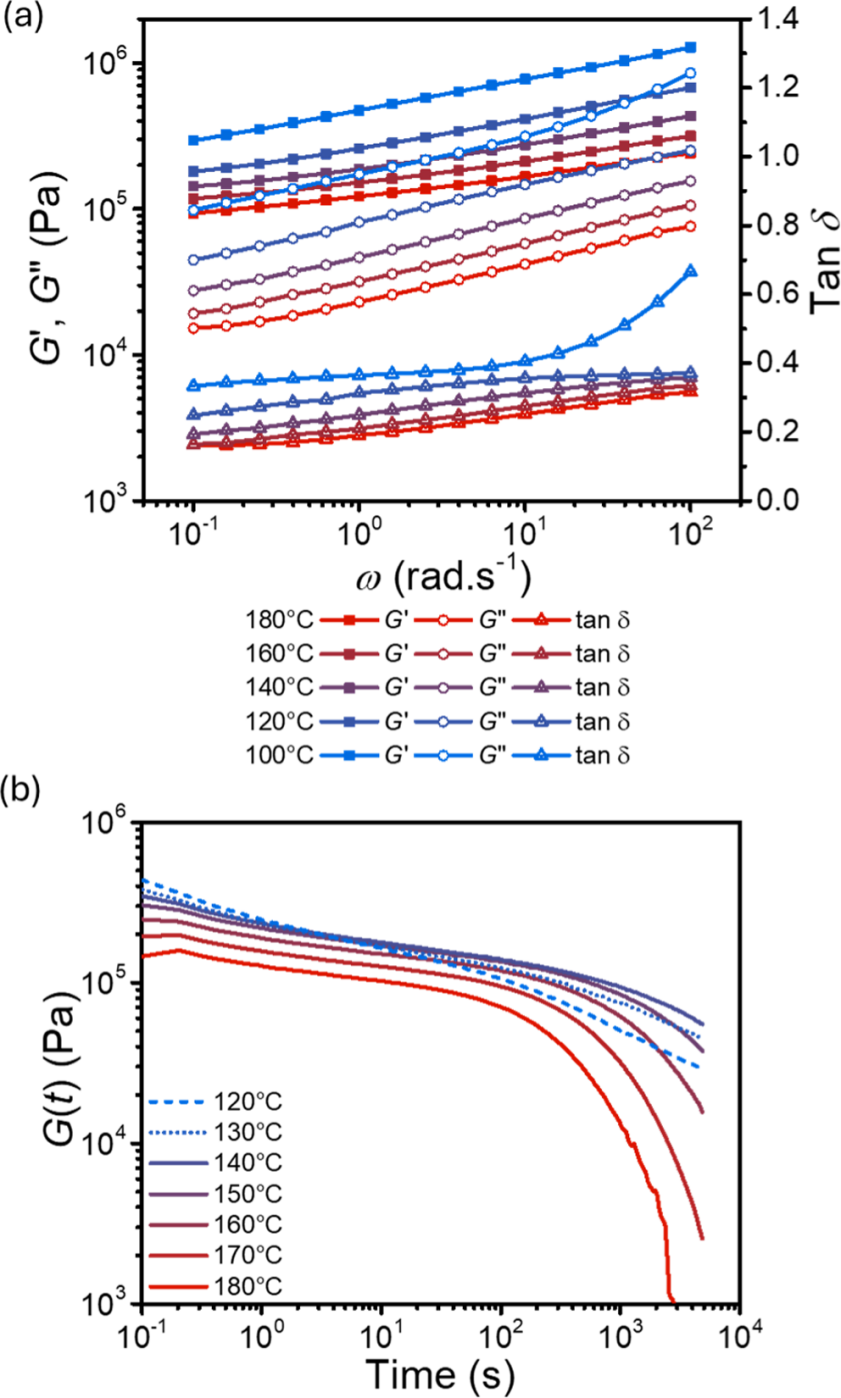
Rheological characterization of **PETG-VU37**. (a) Variable temperature frequency sweeps. The graph shows storage (*G*′) and loss (*G*″) moduli as well as tan δ as a function of angular frequency. The data was collected at a constant strain of γ = 1%. (b) Stress relaxation experiments of **PETG-VU37** from 120 to 180 °C at a constant step strain of γ = 1%. The graph shows the decaying relaxation modulus (*G*(*t*)) as a function of time.

To further probe the dynamic of bond exchange, stress relaxation experiments were conducted on **PETG-VU37** between 120 and 180 °C (Figure 3b and Figure S20). Stress relaxation at 120 and 130 °C is faster than at 140 °C, while relaxation times decrease with temperature from 140 to 180 °C. This unexpected behavior at lower temperatures likely suggests near-*T*_g_ and equilibration effects. Consequently, only data acquired at ≥140 °C was considered in the quantitative analysis. The data reveal at least two distinct relaxation processes: a faster component, likely arising from the segmental dynamics of the PETG chains, and a slower process attributed to the exchange of vinylogous urethane bonds.^84^ The relaxation modulus, *G*(*t*), appears to decrease with increasing temperature (Figure 3b), which is consistent with the frequency sweep and DMA data (Figures 2a and 3b), decreasing from *G*(10 s) of 178 kPa at 140 °C to 102 kPa at 180 °C. To quantify the relaxation behavior of the **PETG-VU37** network, fitting the data with single and double exponential decay equations (Maxwell model) was unsuccessfully attempted. By contrast, the relaxation data is well-described by double stretched exponential decay (double Kohlrausch–Williams–Watts) functions (Figure S20a and Table S3). This implies that two definite relaxation modes with multimodal, nonideal relaxation processes are at play, where the stretching coefficient (β ≤1) indicates the degree of deviation from ideality (in the ideal Maxwell model, β = 1).^66,67,84,85^ The faster of the two relaxation processes could not be reliably quantified using this analysis, since the experiment only captures the tail end of the segmental relaxation. The characteristic relaxation times of the slow process (τ), which were extracted from the double Kohlrausch–Williams–Watts fits, decrease with increasing temperature (from 140–180 °C, Table S3), and an Arrhenius analysis of ln τ vs *T* ^–1^ yields an activation energy (*E*_a_) of 90.8 ± 12.1 kJ mol^–1^ (Figure S20b), which is comparable to the other reported values for vinylogous urethane networks with a similar amine-to-acetoacetate ratio and cross-link density.^62,74^ Interestingly, the stretching coefficient (β) of the slow process becomes closer to 1 as the temperature increases, implying that the system is approaching ideality and the relaxation becomes better defined. At 180 °C, **PETG-VU37** reaches full stress relaxation (residual stress <1%) within ca. 40 min. The integration of the *G*(*t*) vs *t* curve at this temperature yielded a zero-shear viscosity (η_0_) of 4.7 × 10^7^ Pa·s (Figure S20).^39^

To gain further insights into the mechanisms at play during the relaxation of the network, the continuous relaxation spectra (CRS) were extracted from the stress relaxation experiments using the pyReSpect Python code developed by Shanbhag (Figure S21, see Methods section for details).^68^ The continuous relaxation spectrum (CRS) can provide insight into the underlying distribution of relaxation times in stress relaxation experiments, where spectral peaks indicate the dominant relaxation times (relaxation strength as a function of relaxation times) and the feature breadth reflects the extent of the distribution (i.e., deviation from ideality).^86,87^ Here, the relaxation time spectra again show two distinct relaxation processes, a slow one, with well-defined peaks from 150 to 180 °C at higher relaxation times, and a fast one, with less-defined peaks at short time scales (Figure S21). Both processes shift, as expected, toward shorter characteristic times with increasing temperature, and the slow relaxation mode becomes sharper. This is in good agreement with the Kohlrausch–Williams–Watts fits, in which the relaxation nears ideality as the temperature increases, with the stretching coefficient approaching 1 (Table S3). This may indicate that network segmental dynamics become less important and that pure dynamic exchanges of the vinylogous urethanes start to dominate.

Overall, the rheology data indicate that **PETG-VU37** behaves as an incompletely cross-linked network, which is consistent with the relatively low gel fraction and a rubbery plateau with a slope in DMA. Although no crossover between *G*′ and *G*″ is observed, **PETG-VU37** exhibits a relaxation time (τ) of ca. 5.5 min (from fitting) at 180 °C in stress relaxation experiments. This suggests that it is a candidate for a reprocessable material that can be healed quickly, an uncommon feature for associative dynamic covalent networks. Whereas the rheology of **PETG-VU26** indicates that this material is even less cross-linked than **PETG-VU37**, with a viscous liquid behavior at 180 °C, *G*″ > *G*′ in almost all the probed frequency range, this is likely the reason for the observed crystallization at 160 °C and below, essentially decreasing the likelihood that **PETG-VU26** heals effectively.

### Recycling of **PETG-VU** and Impact of Moisture

Due to the limited thermomechanical stability of **PETG-VU71** and the crystallization-prone nature of **PETG-VU26**, recycling and environmental resistance experiments were conducted exclusively with **PETG-VU37**, which demonstrated the most favorable combination of mechanical robustness, network integrity, and thermal processability. Mechanical recycling of **PETG-VU37** was performed by cutting the material into small pieces and compression-molding these at 180 °C under a pressure of 4 tons for 10 min. The reprocessed films were characterized by DMA, tensile testing, and FT-IR spectroscopy (Table 3, Figure 4).

**Table 3:** Effect of Recycling and Moisture Exposure on Properties of **PETG-VU37** ^a^

treatment	*T*_g_ (°C)^b^	*E*′ at 25 °C (MPa)^b^	*E*′ at 150 °C (MPa)^b^	failure temp. (°C)^b^	Young’s modulus (MPa)^c^	tensile strength (MPa)^c^	strain at break (%)^c^	toughness (kJ m^–3^)
original	80 ± 2	1069 ± 203	0.60 ± 0.15	207 ± 8	838 ± 19	40 ± 2	6 ± 1	148 ± 28
1× recycled	86 ± 1	929 ± 115	0.43 ± 0.08	205 ± 6	923 ± 52	40 ± 4	6 ± 1	199 ± 25
2× recycled	90 ± 2	1042 ± 387	0.25 ± 0.05	191 ± 4	934 ± 39	32 ± 2	5 ± 1	114 ± 19
80% RH	76 ± 4	1252 ± 189	0.63 ± 0.15	206 ± 7	950 ± 16	33 ± 8	5 ± 1	102 ± 51

a All data represent averages of *n* = 3 individual measurements ±standard deviation.

b Determined by DMA at a heating rate of 3 °C min^–1^.

c Measured by stress–strain experiments at 25 °C with a strain rate of 150% min^–1^.

**Figure 4:**
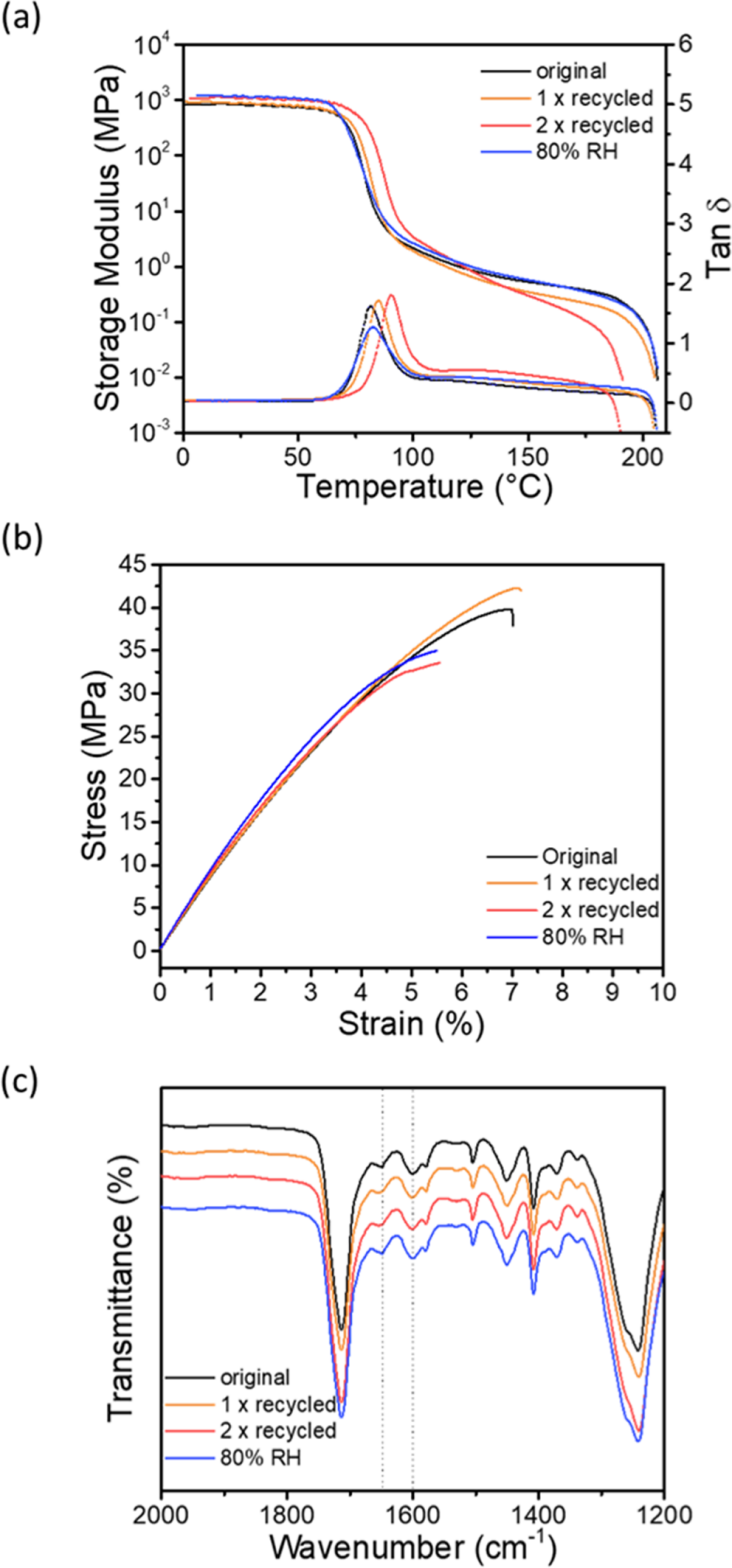
Effect of recycling and moisture exposure on properties of **PETG-VU**. Shown are data for the original **PETG-VU37**, and samples that were 1× or 2× recycled or exposed to 80% relative humidity. (a) Dynamic mechanical analysis (DMA) traces showing the storage modulus *E*′ and loss factor tan δ. (b) Representative stress–strain curves. (c) FT-IR spectra. DMA experiments were carried out at a heating rate of 3 °C min^–1^, and tensile tests were conducted at 25 °C and a strain rate of 150% min^–1^.

After one recycling cycle, **PETG-VU37** retains properties that are similar to those of the pristine material. The *T*_g_ (determined by DMA) increased slightly from 80 to 86 °C, *E*′ (at 25 °C) dropped from 1.07 to 0.93 GPa, the Young’s modulus rose from 0.84 to 0.92 GPa, while the failure temperature (205 °C), tensile strength (40 MPa), and strain at break (6%) are unchanged (Figure 4a,b). Signs of degradation become evident after a second reprocessing cycle. A significant reduction in tensile strength to 32 MPa, a reduction in *E*′ at 150 °C, and a reduced failure temperature (191 °C), along with a darkening of the sample, indicate a decrease in cross-link density, perhaps due to side reactions that affect the dynamic bonds, the free amines, or the polyester backbone, which only happened after prolonged times at 180 °C (two reprocessing cycles).^76,88,89^ However, the FT-IR spectra of the reprocessed samples (Figure 4c) show no major changes compared to the pristine **PETG-VU37**. The characteristic absorption bands associated with the vinylogous urethane carbonyl (1649 cm^–1^) and the alkene stretching mode (1605 cm^–1^) remain unchanged in both position and intensity, indicating that the changes in chemical structure of the dynamic cross-links during reprocessing are indeed minor and cannot be captured by FT-IR spectroscopy. This observation is consistent with the fact that the FT-IR spectra of the parent **PETG-VUs** are all identical.

Because the formation of vinylogous urethane linkages proceeds via an equilibrium reaction that releases water as a byproduct, these bonds are inherently sensitive to moisture.^90–92^ To explore the extent to which moisture impacts the chemical integrity of **PETG-VU37** and its physical properties, samples were exposed to 80% relative humidity at room temperature for 7 days. DMA analysis reveals a modest decrease in *T*_g_ to 76 °C, consistent with modest water uptake that causes a slight plasticization of the polymer. Both *E*′ (at 25 °C) and the Young’s modulus are slightly increased, while strain and stress at break experience a moderate reduction. However, *E*′ measured at 150 °C and the failure temperature remains unchanged, indicating that the cross-link density is, if at all, not significantly impacted, as also confirmed by FT-IR spectroscopy (Figure 4c). While the water uptake leads to some embrittlement, which is also observed when the parent PETG follows the same aging protocol (Figure S22 and Table S4), the vinylogous urethane linkages appear to remain largely intact under humid conditions; even after such exposure, the strength of **PETG-VU37** is on par with that of the parent PETG (Tables 3 and S4).

### Healing of Vinylogous Urethane Networks (**PETG-VU37**)

The healing efficiency of **PETG-VU37** was evaluated to assess the material’s capacity for recovery from damage via thermal activation of the dynamic vinylogous urethane bonds. Samples were deliberately damaged by introducing a controlled cut to approximately 30% of the film’s thickness using a razor blade mounted on a caliper. As expected, the introduction of this defect led to a marked reduction in tensile strength and toughness (Figure 5a and Table 4). Remarkably, thermal treatment at 180 °C for 15 min causes the complete disappearance of the cut, without any noticeable deformation of the film (Figure 5b). Mechanical tests confirm that this healing process effectively restores the material’s performance. The toughness was fully recovered, and 95% of the original tensile strength was regained (Figure 5a and Table 4). These results underscore the excellent healing capability of **PETG-VU37** and confirm the functional efficiency of the dynamic vinylogous urethane chemistry within this network architecture.

**Figure 5:**
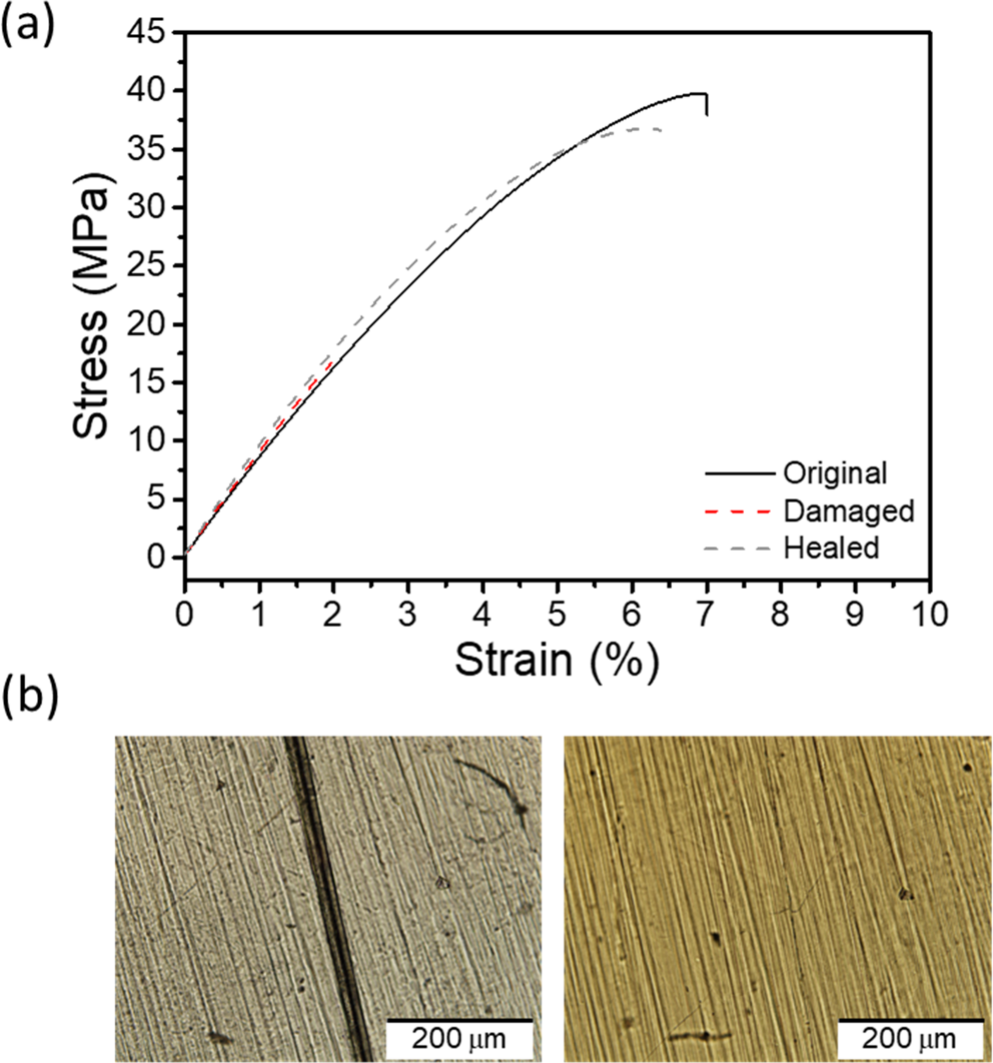
Healing of **PETG-VU37** films. (a) Representative stress–strain curves of pristine, damaged, and healed films, recorded at 25 °C and a strain rate of 150 mm s^–1^. (b) Optical microscopy images of a scratched film (left) and after healing at 180 °C for 15 min (right).

**Table 4:** Healing of Scratched **PETG-VU37** Films^a^

sample	Young’s modulus (MPa)	tensile strength (MPa)	strain at break (%)	toughness (kJ m^–3^)	healing efficiency (%)^b^
original	838 ± 19	40 ± 2	6 ± 1	148 ± 28	n.a.
damaged^c^	940 ± 33	17 ± 6	2 ± 1	20 ± 13	n.a.
healed^d^	981 ± 15	38 ± 3	6 ± 1	148 ± 52	100 ± 3

a Data represents averages of *n* = 3 individual measurements ±standard deviation.

b The healing efficiency is expressed as the ratio of the toughness of the healed and the original samples. The error was calculated by error propagation.

c Damaged samples were scratched to a depth of around 30% of the original sample thickness using a razor blade attached to a caliper for precise depth control.

d Healed samples were exposed to 180 °C for ca. 15 min until the scratch disappeared.

## Conclusions

In summary, we developed a scalable and sustainable strategy to synthesize high-performance, healable vinylogous urethane networks from commercially available PETG. By combining a straightforward depolymerization and functionalization approach with dynamic covalent cross-linking, we produced PETG-based networks that exhibit high tensile strengths of up to 40 MPa and remarkable self-healing behavior. **PETG-VU37**, the most robust formulation, recovered 95% of its original strength following a brief thermal treatment at 180 °C, demonstrating the efficacy of the dynamic bond exchange. In addition to being reprocessable, these materials showed promising environmental resistance and retained their functional properties under moderate humidity. This work highlights the potential of polyester-based dynamic covalent networks as a platform for sustainable, reprocessable, and healable materials. We speculate that combining vinylogous urethanes with supramolecular motifs may further increase the healing efficiency and expand the functional scope of these adaptable polymer systems.

## Supplementary Material



## Notes

^1^H NMR spectra, SEC traces, FTIR, additional DMA, TGA and DSC traces, rheology data, tensile and DMA data for the parent PETG (pristine and aged), and tables with additional mechanical data (PDF)
