# Supplementary material for: Healable and Reprocessable PETG-Based Dynamic Vinylogous Urethane Networks
Source: Macromolecules. 2026 Feb 13;59(4):2567–79. doi: 10.1021/acs.macromol.5c02738 (PMC12947682; doi:10.1021/acs.macromol.5c02738)
Supplement: Supplementary file 1 [file ma5c02738_si_001.pdf]

Supplementary Information

Healable and reprocessable PETG-based dynamic vinylogous urethane networks

Chaninya Mak-Iad,[1,2] *José Augusto Berrocal,*[1,3,4] *Georges J.M. Formon,*[1,2]* *Christoph Weder*[1,2]*

[1]Adolphe Merkle Institute, University of Fribourg, Chemin des Verdiers 4, 1700 Fribourg, Switzerland

[2]NCCR Bio-inspired Materials, University of Fribourg, Chemin des Verdiers 4, 1700 Fribourg, Switzerland

[3]Institute of Chemical Research of Catalonia (ICIQ), Barcelona Institute of Science and Technology (BIST), Av. Països Catalans, 16, Tarragona, E-43007, Spain

[4]ICREA, Pg. Lluís Companys 23, 08010 Barcelona, Spain.

*Corresponding Authors: georges.formon@unifr.ch; christoph.weder@unifr.ch

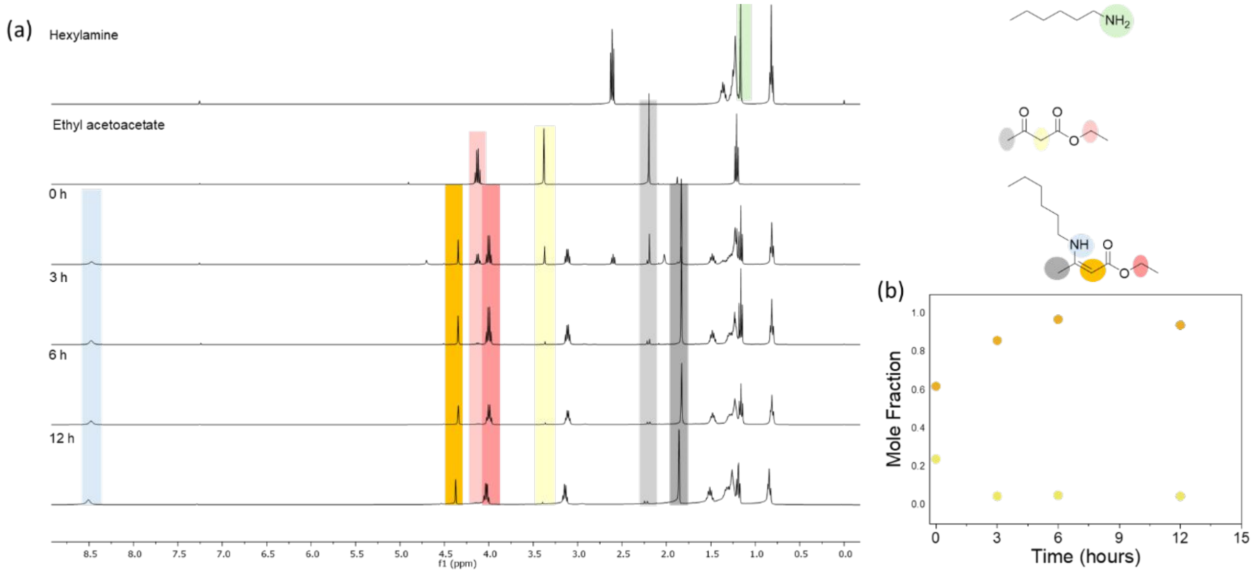

Figure S1. (a) ^{1}H NMR spectra collected over the course of 12 h during the model reaction between hexylamine and ethyl acetoacetate (**Scheme 1** in the main manuscript). Diagnostic signals are color-coded. The spectra reveal the consumption of the starting materials and the concurrent, exclusive formation of the vinylogous urethane product. Full conversion is reached after 6 hours. (b) Plot of the mol-fraction of acetoacetate (yellow circles) and vinylogous urethane (orange circles) as a function of time. These values were obtained by comparing the integrals of the protons depicted in orange/yellow.

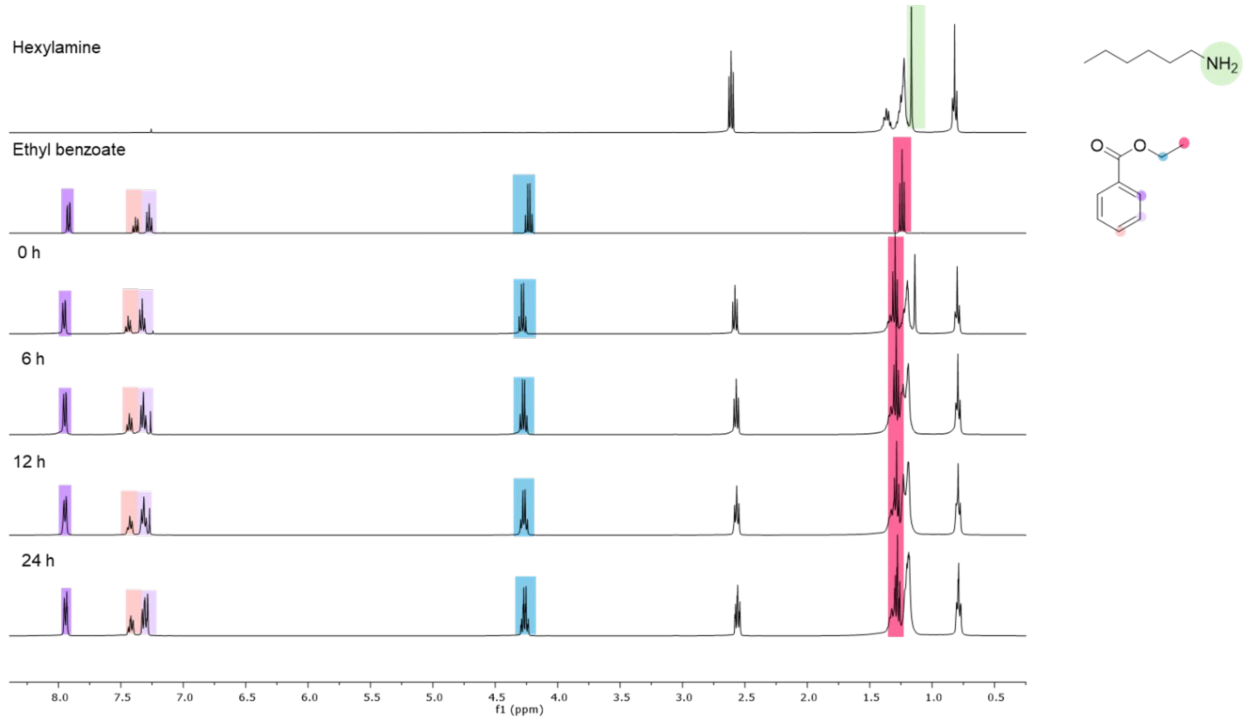

Figure S2. ^{1}H NMR spectra collected over the course of 24 h during the model reaction between hexylamine and ethyl benzoate (**Scheme 1** in the main manuscript). The absence of any significant changes in the spectra indicates that the two compounds do not react under these conditions.

(a)

(b)

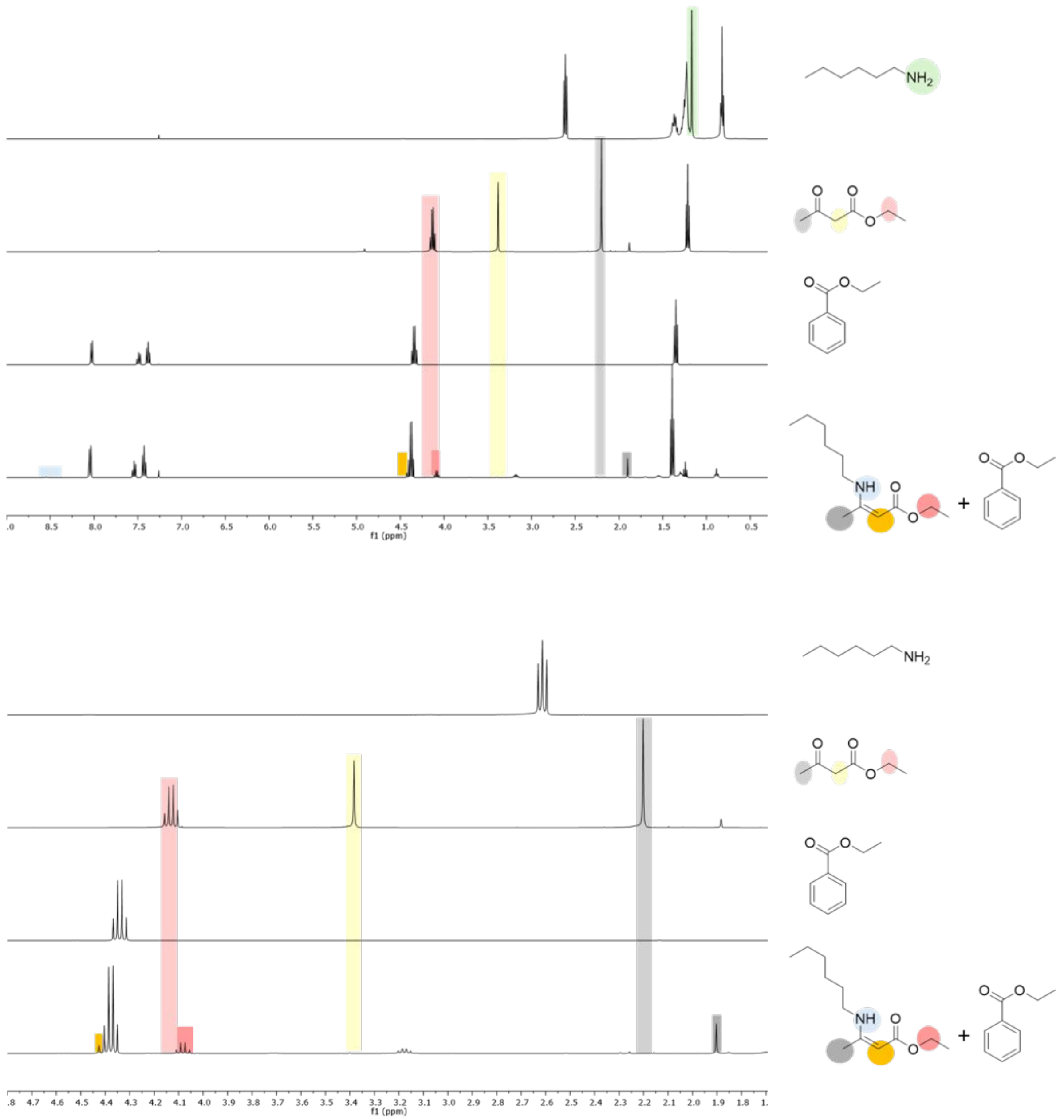

Figure S3. ^{1}H NMR spectra collected after 24 h for the model reaction between hexylamine and a 1:9 mol/mol mixture of ethyl acetoacetate and ethyl benzoate (**Scheme 1** in the main manuscript). (a) Full spectrum, (b) magnification of a range with pertinent signals. A comparison with the 12 h spectrum shown in **Figure S1** shows that the only reaction product is the VU, while the slight excess of hexylamine and the ethyl benzoate remain unreacted under these conditions.

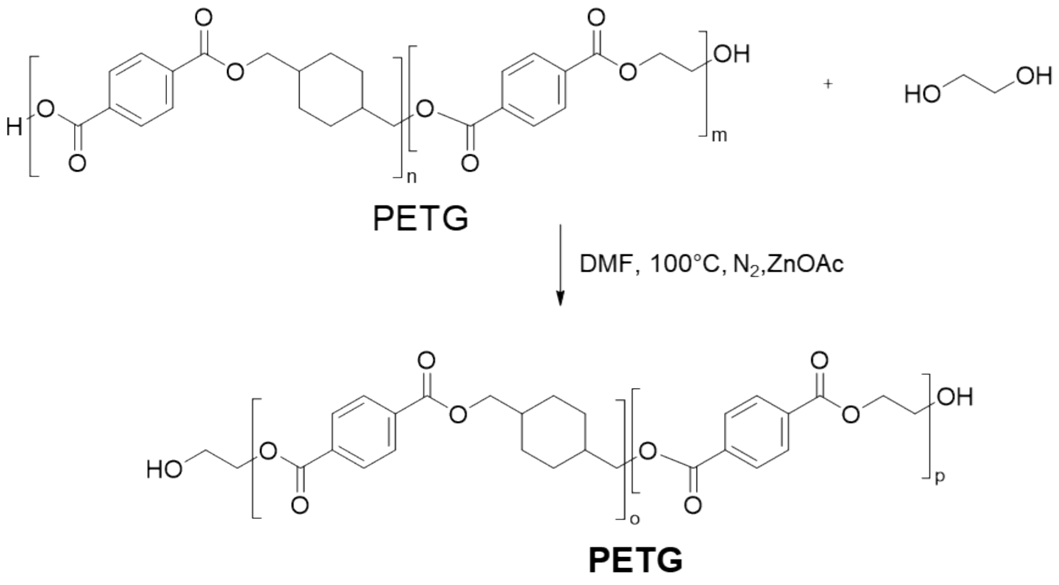

Scheme S1. Synthesis of the hydroxy-terminated telechelic **PETG-OH.**[1]

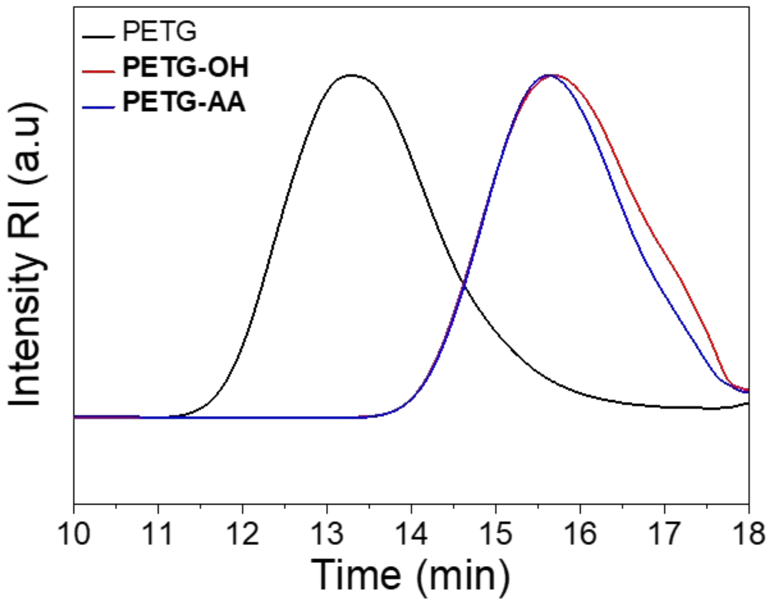

Figure S4. Representative size exclusion chromatography (SEC) elugrams of a commercial PETG, the hydroxyl-terminated PETG telechelic (**PETG-OH**), and PETG-bis-acetoacetate (**PETG-AA**). The chromatograms were recorded using a refractive index (RI) detector.

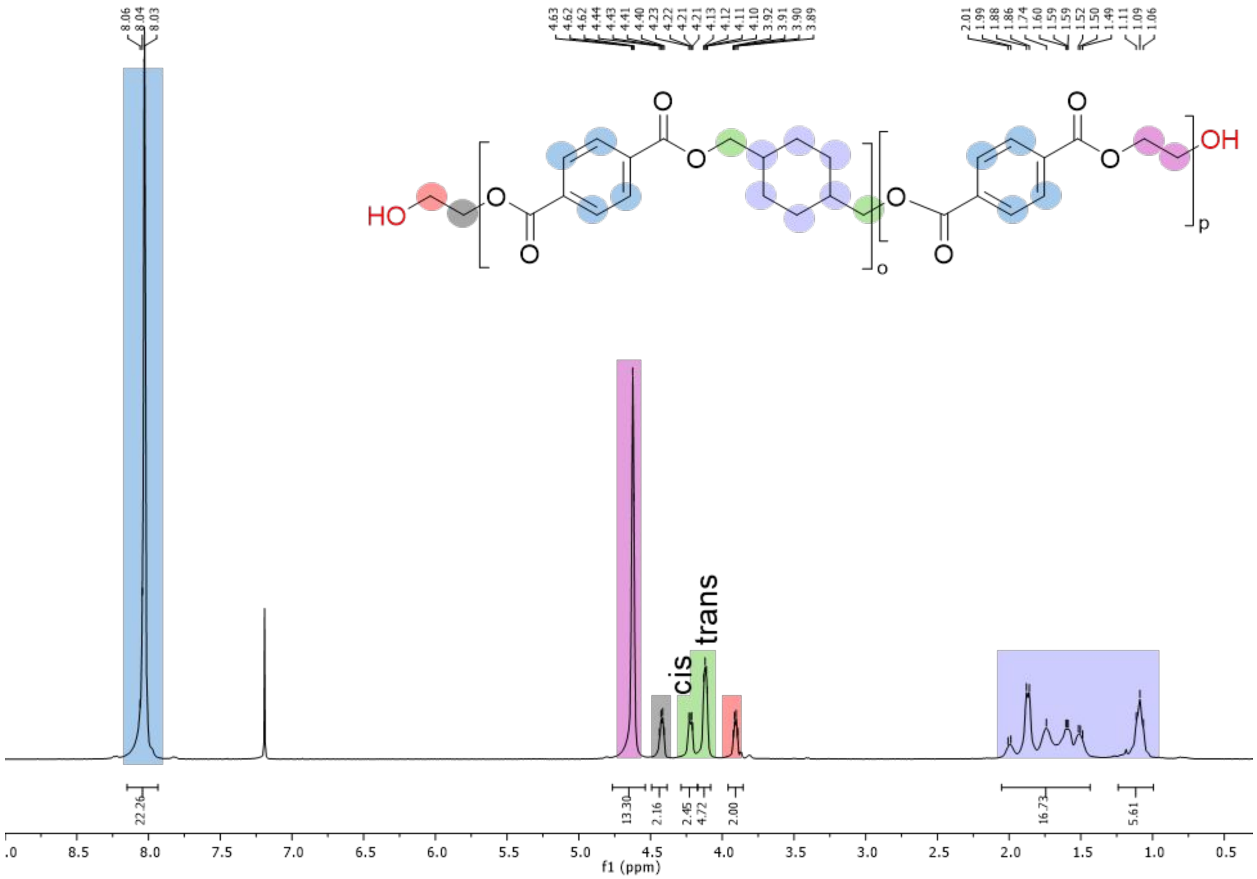

Figure S5. ^{1}H NMR spectrum ($CDCl_3$, 400 MHz) of **PETG-OH**. The peaks at 8.04 ppm (main-chain) and at 4.4 and 3.9 ppm (end-groups) were used for calculating M_n.

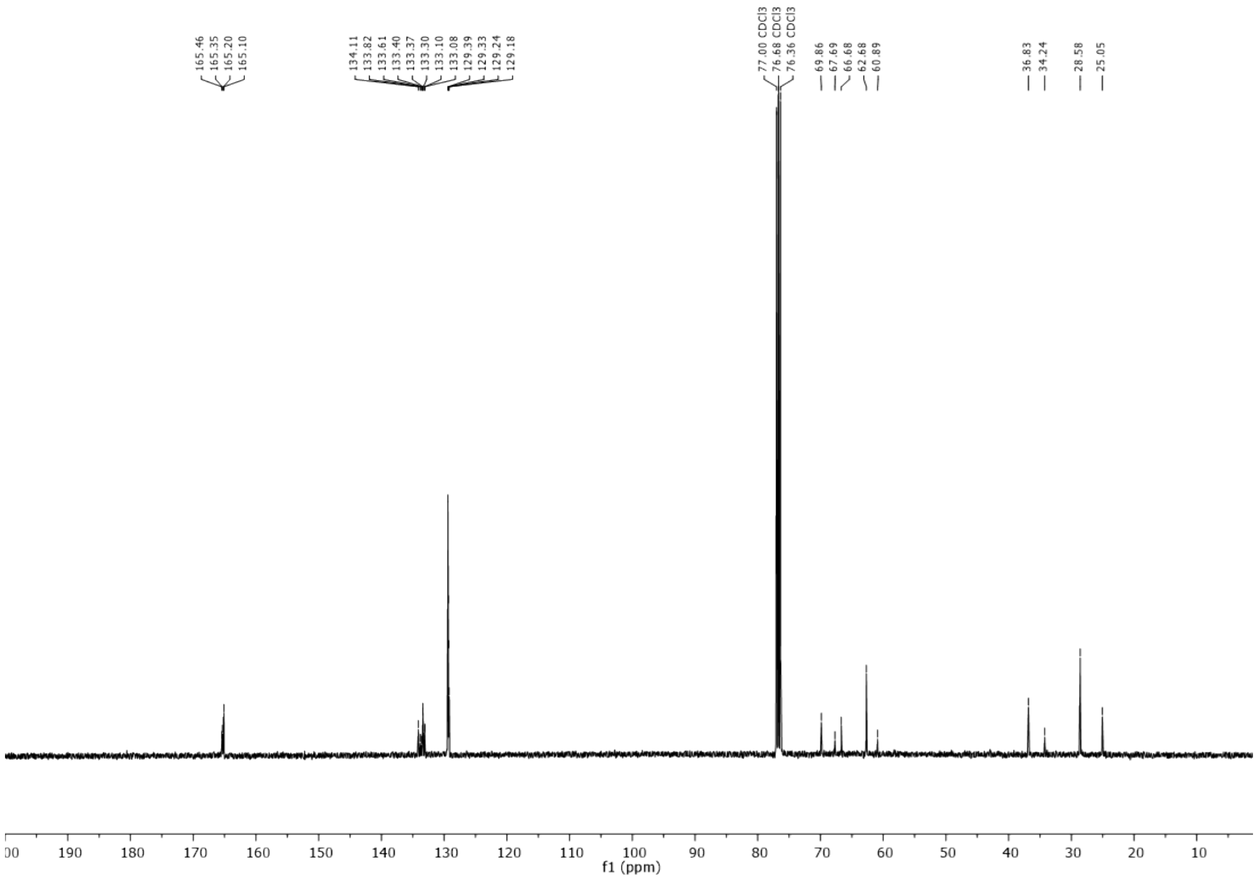

Figure S6. ^{13}C NMR spectrum ($CDCl_3$, 101 MHz) of **PETG-OH**.

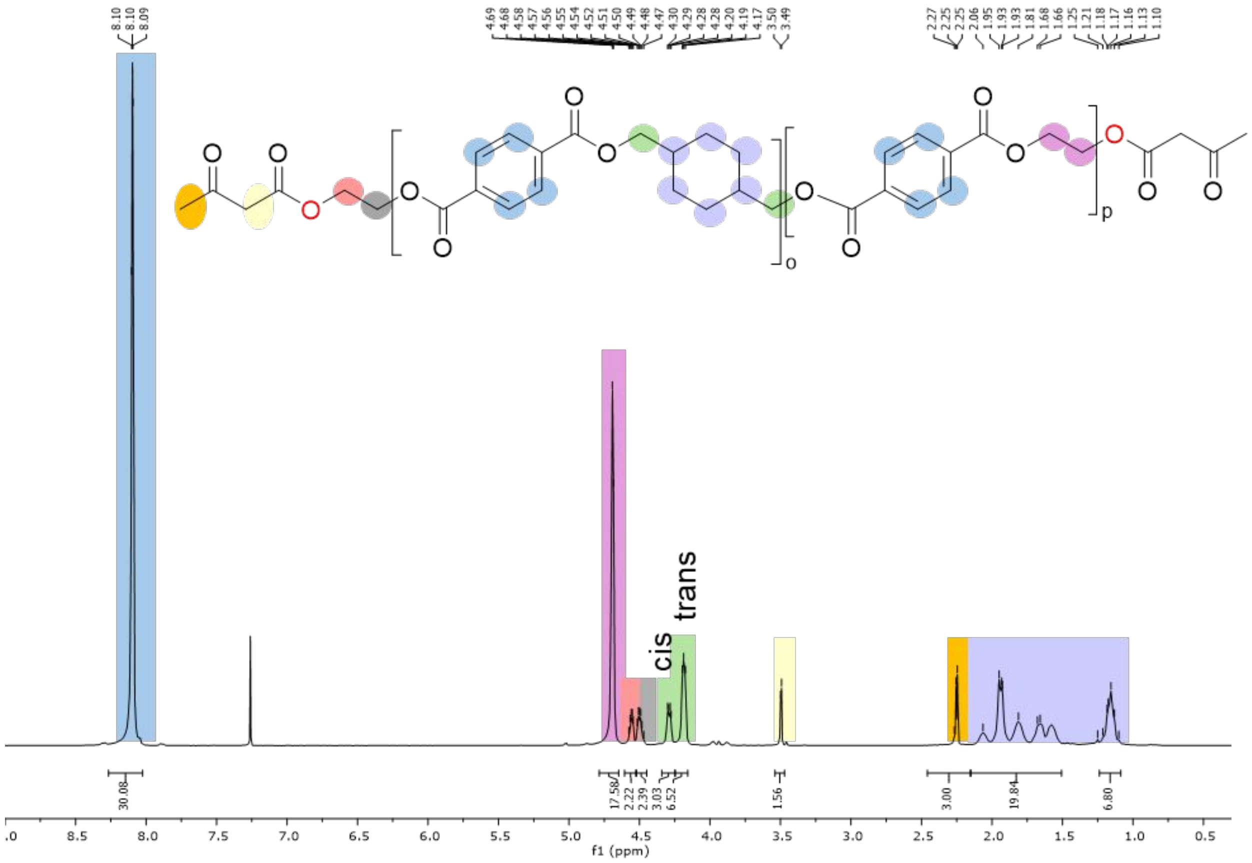

Figure S7. ^{1}H NMR spectrum ($CDCl_3$, 400 MHz) of **PETG-AA**. The peaks at 8.04 ppm (main-chain) and at 3.5 and 2.25 ppm (end-groups) were used for calculating M_n.

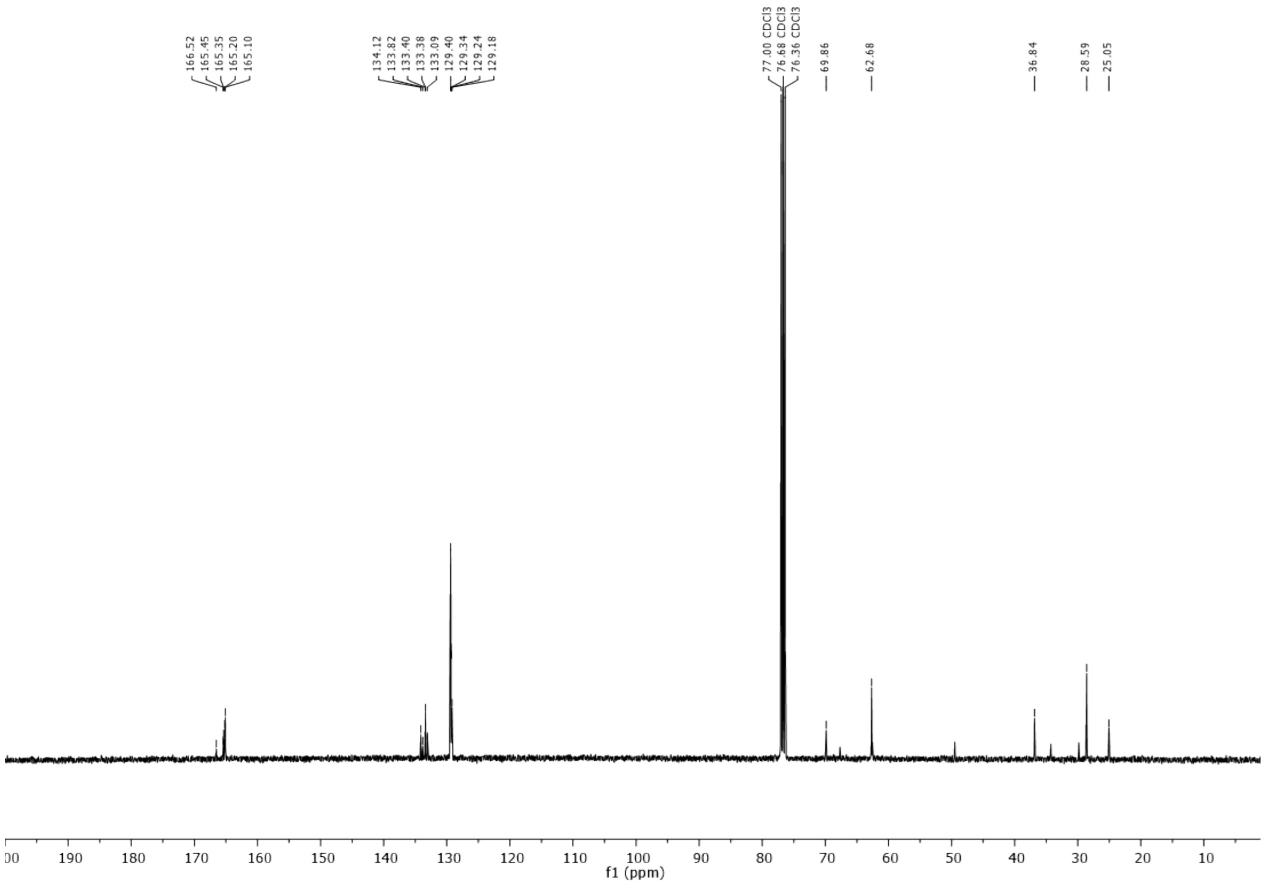

Figure S8. ^{13}C NMR spectrum ($CDCl_3$, 101 MHz) of **PETG-AA**.

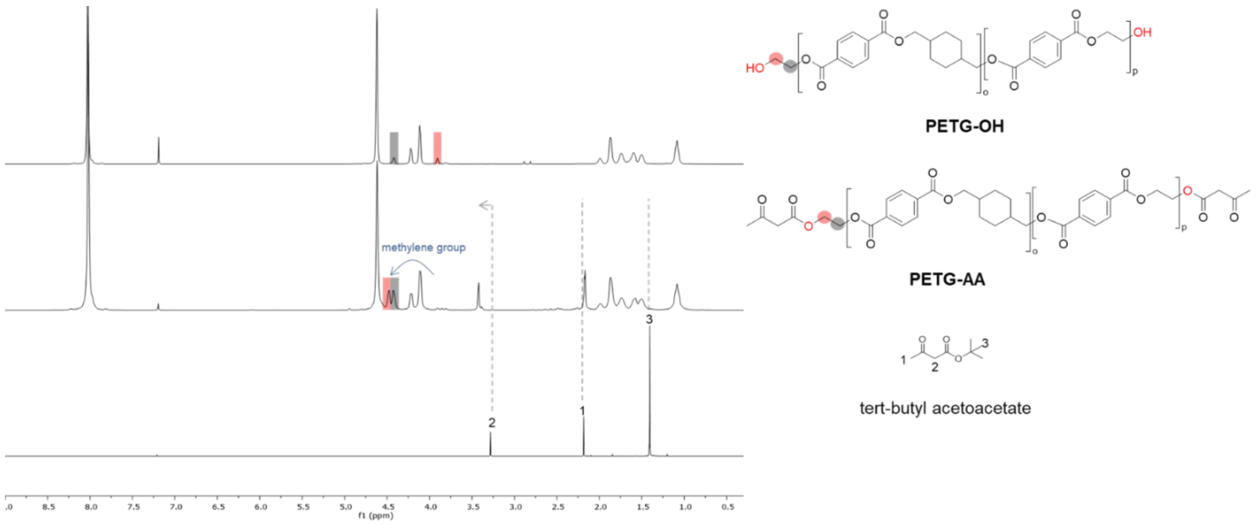

Figure S9. ^{1}H NMR spectra ($CDCl_3$, 400 MHz) of **PETG-OH**, **PETG-AA**, and tert-butyl aceto-acetate.

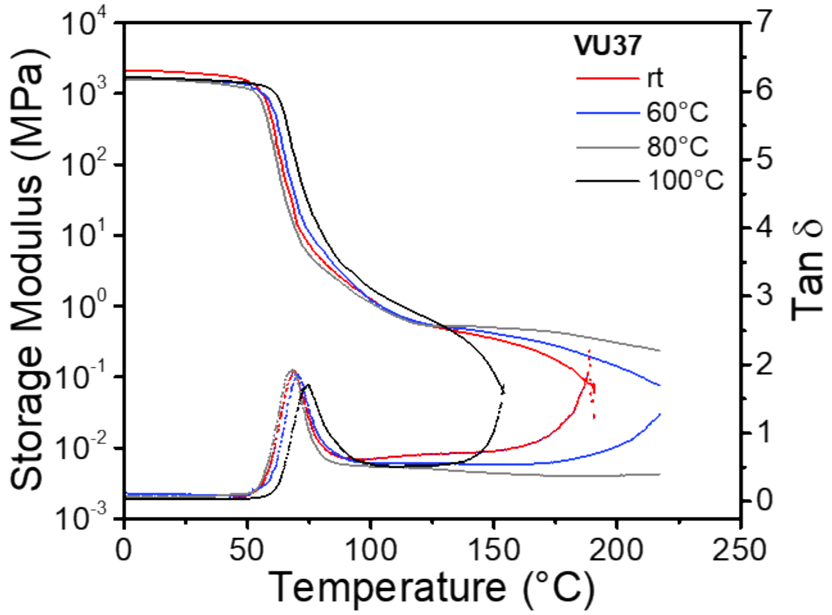

Figure S10. Mechanical properties of **PETG-VU37** prepared using different drying temperatures. Except for the drying temperature, the procedure was the same for all samples, as described in the methods section (after drying, the networks were cured at 100 °C under vacuum for 12 hours and subsequently compression-molded at 180 °C for 10 minutes).

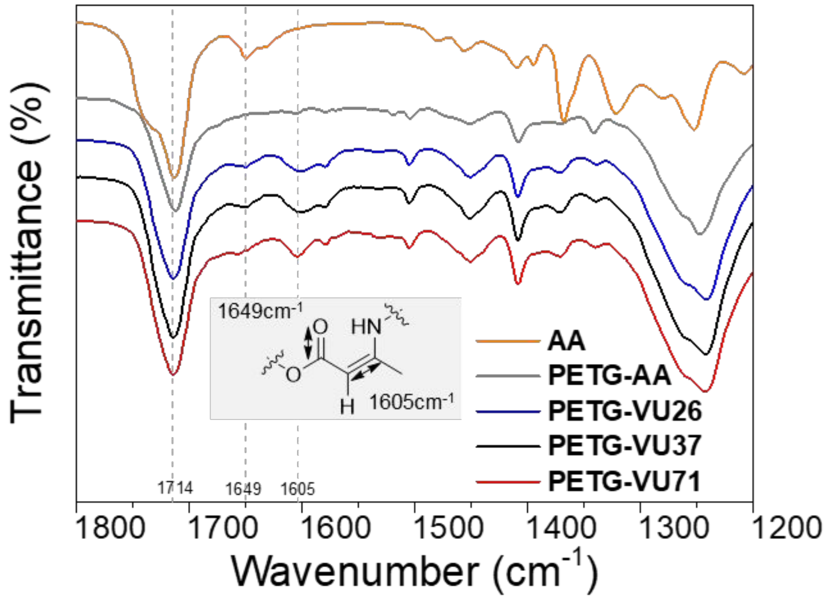

Figure S11. FT-IR spectra of tert-butyl acetoacetate, **PETG-AA**, and the different **PETG-VU** samples. The characteristic stretching vibrations of the carbonyl groups, specifically those corresponding to the esters, are observed at 1714 cm^{-1}. The gray box highlights the stretching vibrations of the vinylogous urethane carbonyl (1649 cm^{-1}) and double bonds (1605 cm^{-1}). These assignments correspond to the characteristic bands of the vinylogous urethane groups.

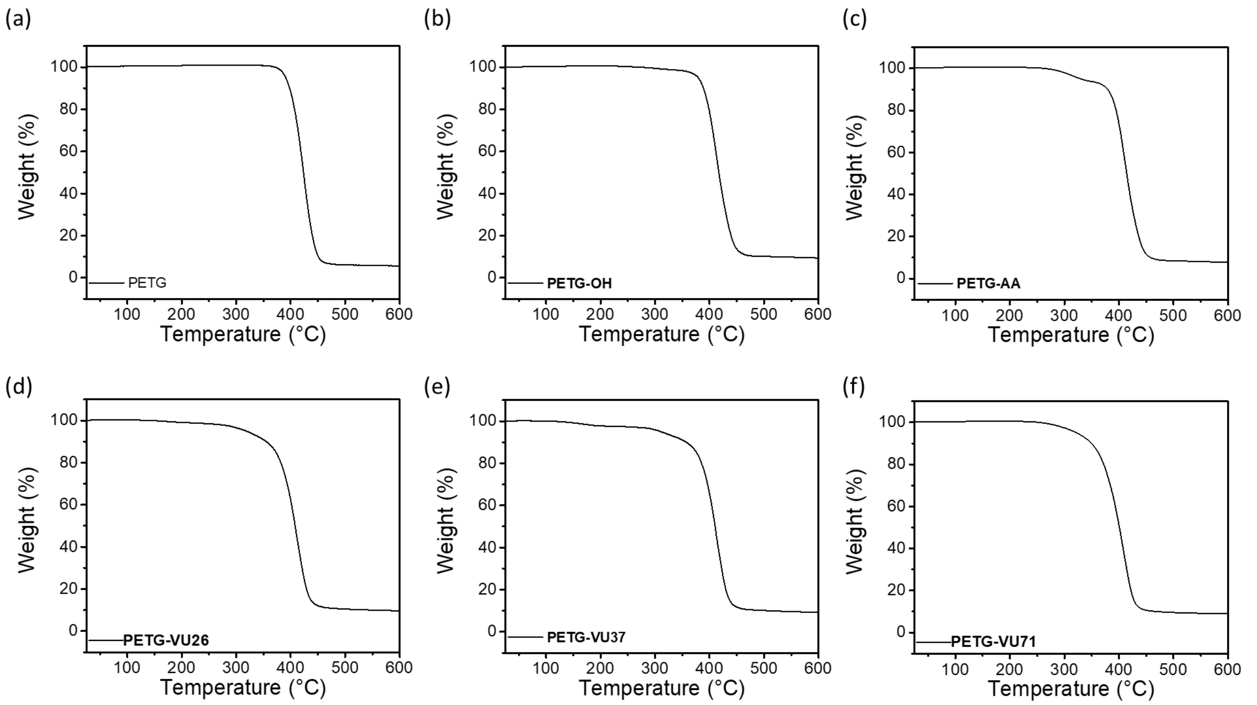

Figure S12. Thermogravimetric analysis (TGA) traces of (a) PETG, (b) **PETG-OH**,(c) **PETG-AA**, (d) **PETG-VU26**, (e) **PETG-VU37**, and (f) **PETG-VU71**.

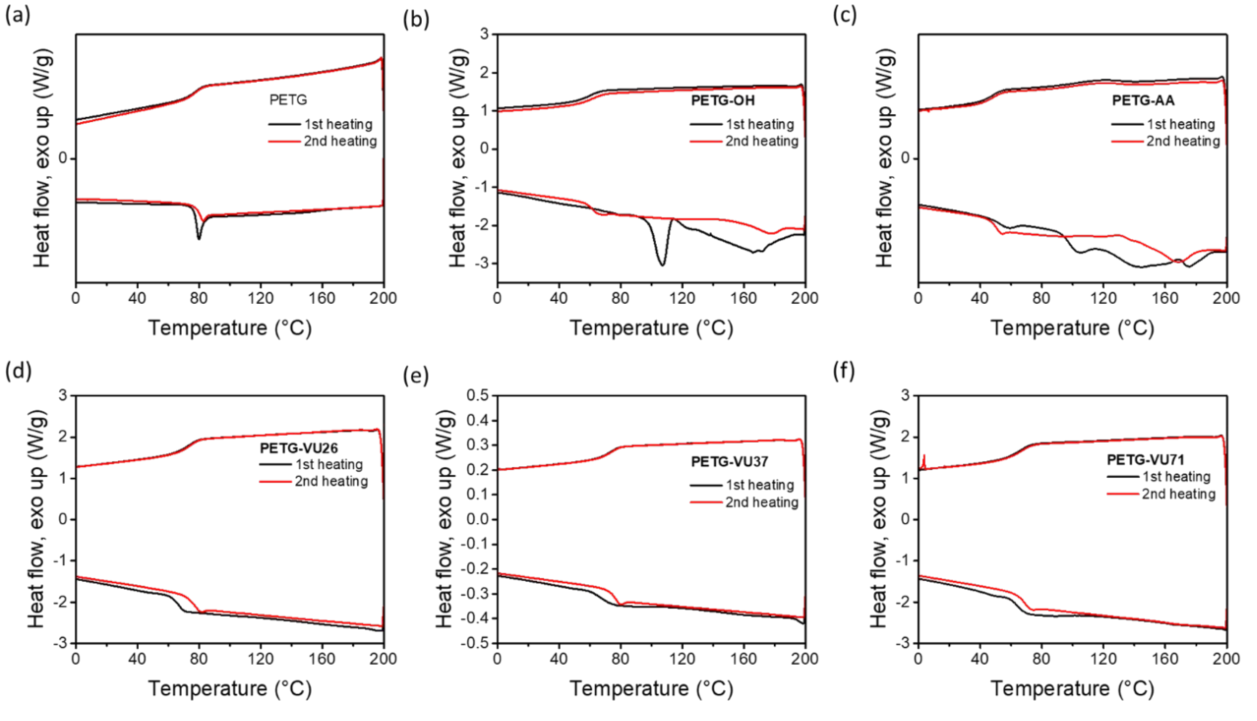

Figure S13. Differential scanning calorimetry (DSC) traces of (a) PETG, (b) **PETG-OH**, (c) **PETG-AA**, (d) **PETG-VU26**, (e) **PETG-VU37**, and (f) **PETG-VU71**. The vinylogous urethanes had been freshly melt-processed at 180°C for 10 min just before the DSC experiments.

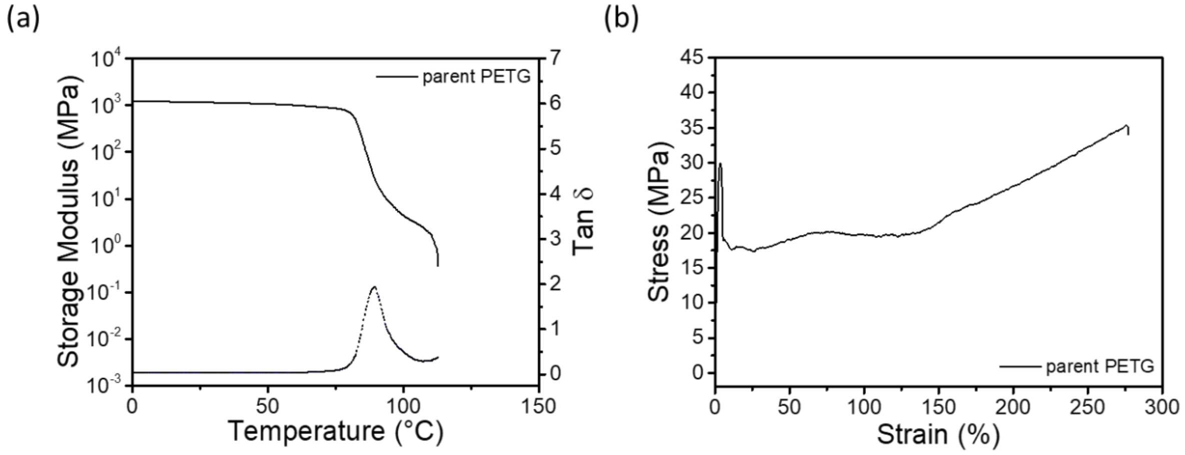

Figure S14. Mechanical properties of the parent PETG. a) Dynamic mechanical analysis (DMA) traces showing the storage modulus E' and the loss factor tan δ. (b) Representative stress-strain curve. The DMA experiments were carried out at a heating rate of 3°C min^{-1}, and tensile tests were conducted at 25°C and a strain rate of 150% min^{-1}.

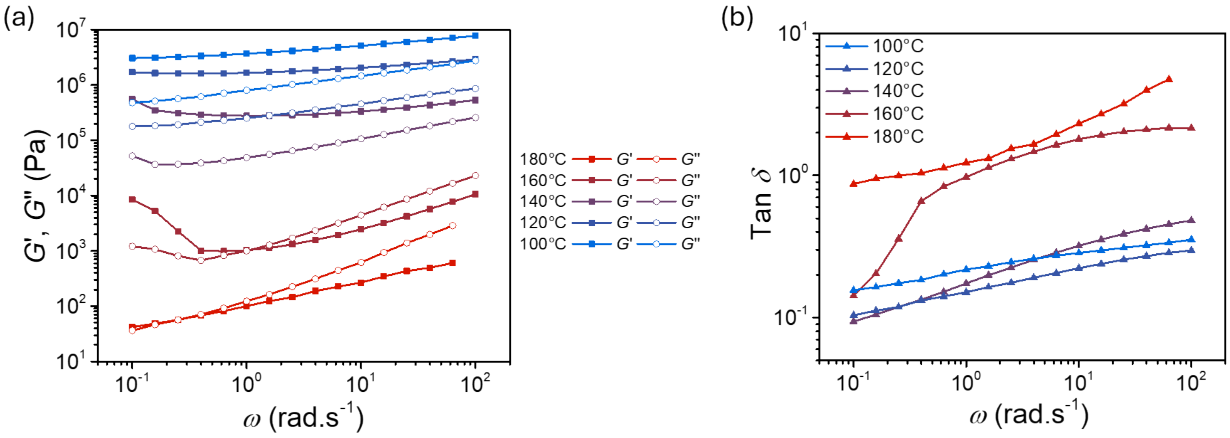

Figure S15. Small-angle oscillatory shear (SAOS) frequency sweeps of **PETG-VU26** at constant deformation γ = 1%. Shown are (a) storage modulus and loss modulus, and (b) tan δ traces as a function of angular frequency for different temperatures. The multi-temperature frequency sweeps were performed by cooling the sample from 180 °C (see Experimental Section for Details).

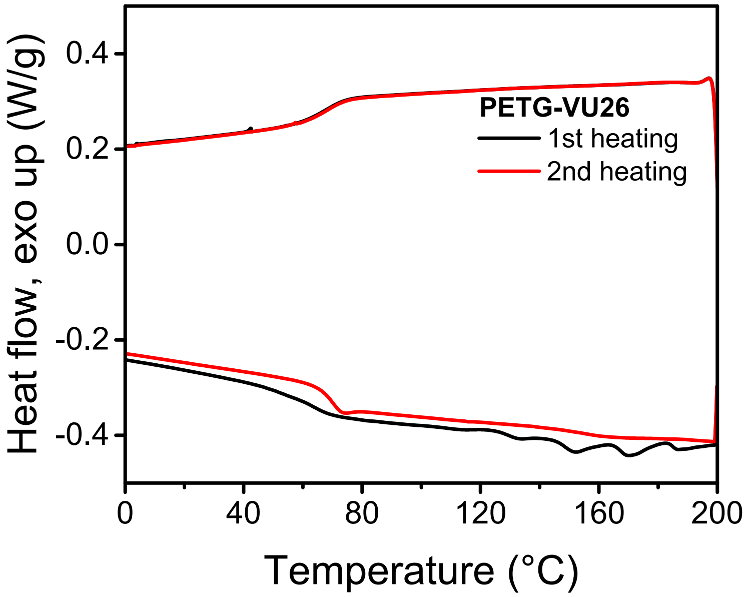

Figure S16. Differential scanning calorimetry (DSC) traces of **PETG-VU26** after the rheology experiment. The DSC trace reveals weak melting peaks that are indicative of crystallization.

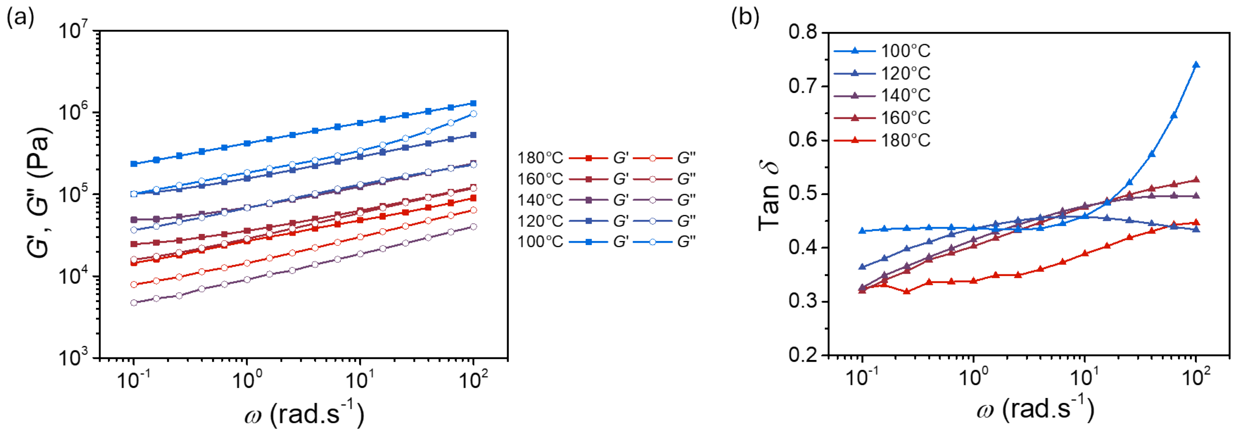

Figure S17. Small-angle oscillatory shear (SAOS) frequency sweeps of **PETG-VU37** at constant deformation $\gamma = 1\%$. Shown are (a) storage modulus and loss modulus, and (b) tan δ traces as a function of angular frequency for different temperatures. The multi-temperature frequency sweeps were performed by cooling the sample from 180 °C (see Experimental Section for Details).

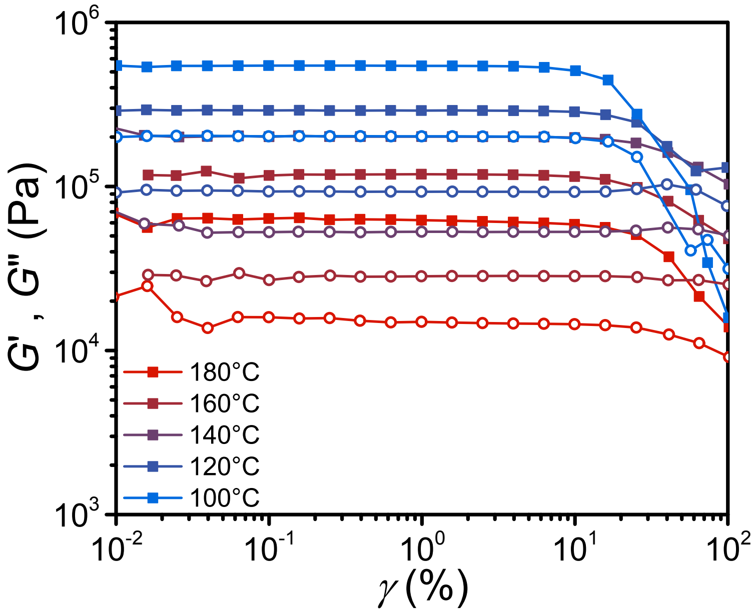

Figure S18. Multi-temperature amplitude sweeps of **PETG-VU37** at a constant angular frequency of $\omega = 1$ rad·s^{-1}. The samples start to deviate from the linear regime above 10% strain.

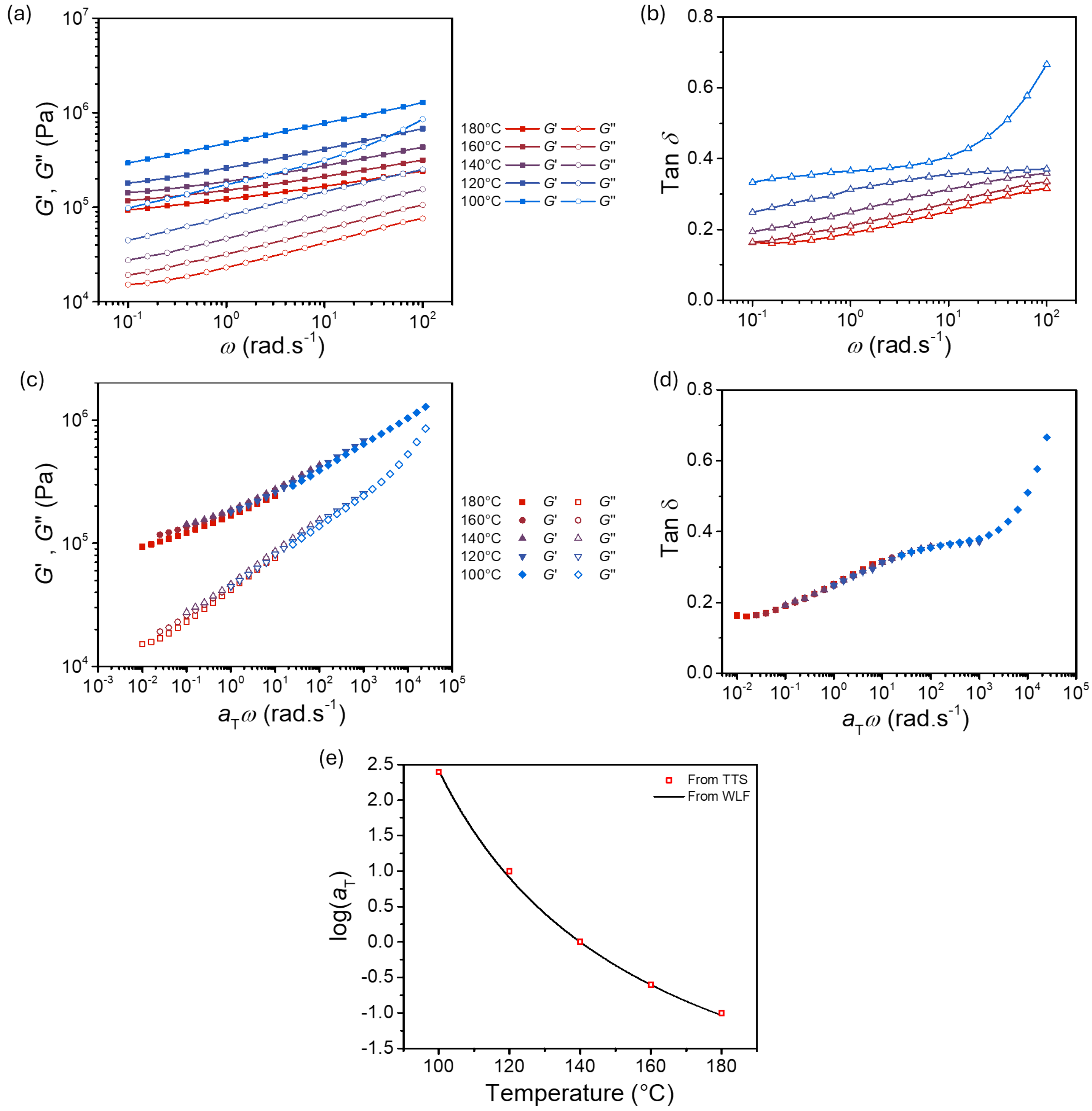

Figure S19. Small-angle oscillatory shear (SAOS) frequency sweeps of **PETG-VU37** at constant deformation γ = 1%. Shown are (a) storage modulus and loss modulus, and (b) tan δ traces as a function of angular frequency for different temperatures. The multi-temperature frequency sweeps were performed by heating the sample from 100 to 180°C (see Experimental Section for Details). (c) Storage and Loss moduli and (d) tan δ master curves made using the time-temperature superposition principle with a reference temperature of 140°C using the data from (a) and (b), which were shifted horizontally. (e) Logarithm of horizontal shift factors used for the creation of the master curve in (c) and (d), plotted as a function of temperature (squares) and the Williams–Landel–Ferry fit (solid line), where C_1 = 3.61 and C_2 = 99.8 K (R^2 = 0.998).

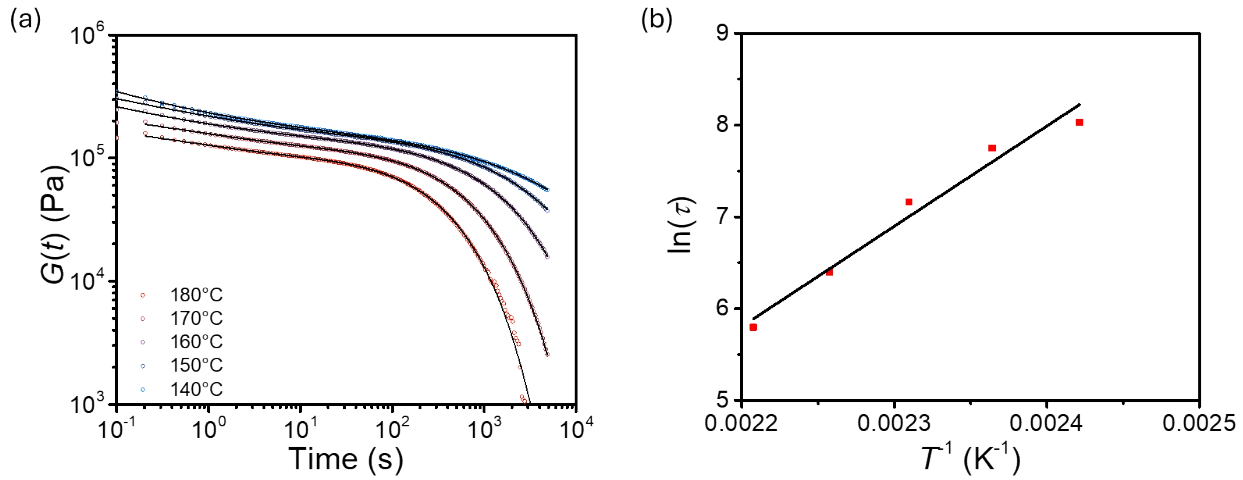

Figure S20. Rheological stress relaxation experiments of **PETG-VU37** at constant deformation γ = 1% and different temperatures. (a) Log-log plots of the relaxation modulus as a function of time. The hollow circles represent the data, while the solid lines represent the fits to a double stretched exponential decay model ($R^2 \geq 0.9999$; see the Methods section for details, and Table S3 for results). (b) Plot of the natural logarithm of relaxation times as a function of the inverse temperature. The red squares represent the 'slow' relaxation time value by the fitting of a stretched double exponential decay (see Methods section for details, Table S3), while the straight line is the Arrhenian fit (E_a = 90.8 ± 12.1 kJ.mol^{-1}, R^2 = 0.963, the error represents the standard error of the fit).

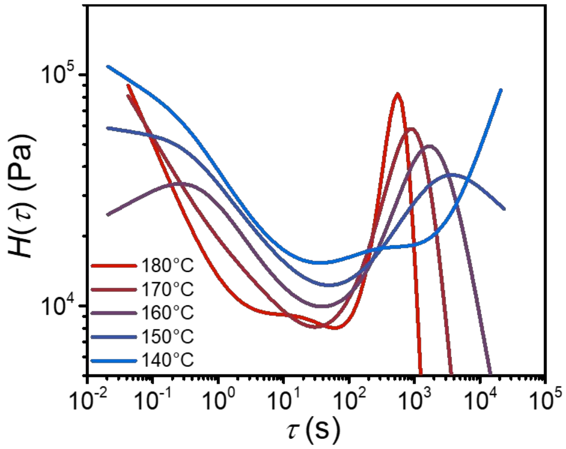

Figure S21. Continuous relaxation spectra of **PETG-VU37** from stress relaxation experiments at different temperatures. Log-log plots of the relaxation time spectra, $H(\tau)$, as a function of relaxation times (τ).

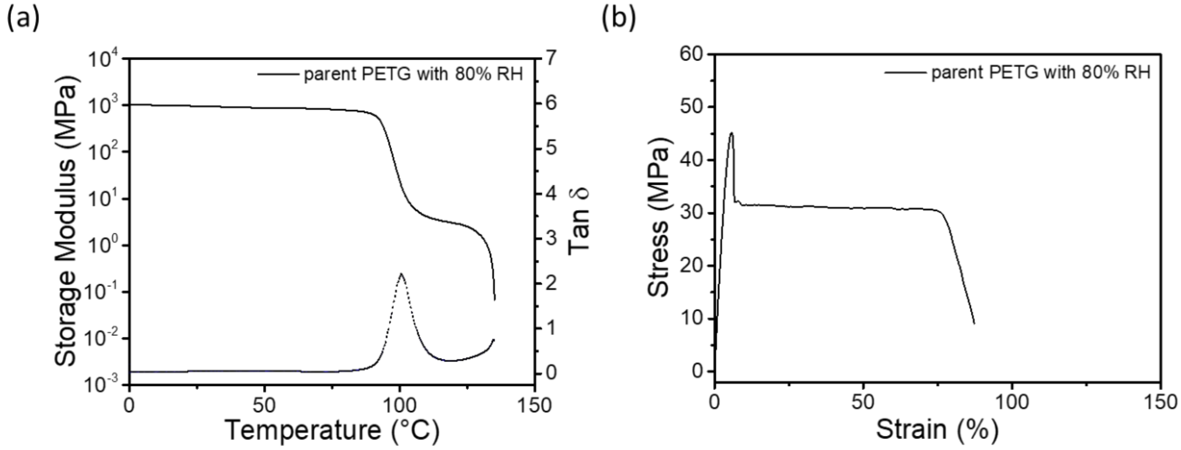

Figure S22. Mechanical properties of the parent PETG after conditioning at 80% RH for 7 days. a) Dynamic mechanical analysis (DMA) traces showing the storage modulus *E*' and the loss factor tan δ. (b) Representative stress-strain curve. The DMA experiments were carried out at a heating rate of 3° min^{-1}, and tensile tests were conducted at 25 °C and a strain rate of 150% min^{-1}.

Table S1. Molecular weights of PETG, **PETG**, and **PETG-AA**.

	$M_{n,NMR}$ (kg mol^{-1})[a]	$M_{n,SEC}$ (kg mol^{-1})[b]	$Đ$ (-)[b]
PETG	n.d.[c]	30	1.6
**PETG-OH**	2.4	4.2	1.6
**PETG-AA**	3.1	4.7	1.5

[a]Number-average molecular weight determined by ^{1}H NMR end group analysis.

[b]Number-average molecular weight determined by size exclusion chromatography (SEC) based on poly(styrene) standards.

[c]Not determined, because the peaks of the end groups are not well-resolved in the high-molecular-weight polymer.

Table S2. Swelling ratio and gel fraction of **PETG-VU** samples.

Sample	Solvent	Swelling ratio (%)[a]	Gel Fraction (%)[a]
**PETG-VU26**	THF	327±24	51±4
	DMF	132±8	74±9
**PETG-VU37**	THF	276±35	63±2
	DMF	94±17	81±5
**PETG-VU71**	THF	648±35	30±6
	DMF	338±22	56±1

All data represent averages of n = 3-4 individual measurements ± standard deviation.

[a]After immersion for 24 hours in solvent.

Table S3. Results of the fits of double stretched exponential decay models to the stress relaxation data and the continuous relaxation spectra (CRS) of **PETG-VU37**.

Temperature (°C)	τ_{slow} (s) fit[a]	β_{slow} fit[a]	τ_{slow} (s) CRS[b]
140	3070	0.36	-[c]
150	2322	0.50	3756
160	1292	0.59	1614
170	599	0.65	949
180	329	0.68	556

[a]Obtained from the fit of the double Kohlrausch–Williams–Watts model to the stress relaxation data ($R^2 \geq 0.9999$).

[b]Obtained from the spectral peaks of the continuous relaxation spectra.

[c]Not found.

Table S4. Thermal and mechanical properties of the parent PETG before and after exposure to 80% RH for 7 days.

Sample	T_g (°C)[a]	*E*' at 25°C (MPa)[a]	Failure temp. (°C)[a]	Young's Modulus (MPa)[b]	Yield Stress (MPa)[b]	Tensile strength (MPa)[b]	Yield point (%)[b]	Strain at break (%)[b]
Parent PETG	88±2	1135±121	115±6	1489±72	36±5	33±4	4±0.3	242±48
80% RH aged PETG	99±3	1206±224	132±3	1183±46	45±1	45±1	6±0.5	42±39

All data represent averages of n = 3 individual measurements ± standard deviation.

[a]Measured by DMA.

[b]Measured by stress-strain experiments at 25°C with a strain rate of 150% min^{-1}.

Supporting References

1. C. Mak-iad, L. Bertossi, G. J. M. Formon and C. Weder, *ACS Macro Letters*, 2025, **14**, 996-1003.
